# Syntheses, structures and anti­cancer activities of Cu^II^ and Zn^II^ complexes containing 1,1′-[(3-fluoro­phen­yl)methyl­ene]bis­[3-(3-fluoro­phen­yl)imidazo[1,5-*a*]pyridine]

**DOI:** 10.1107/S2056989024011617

**Published:** 2025-01-01

**Authors:** Hai Le Thi Hong, Hau Nguyen Van, Duong Hoang Tuan, Hung Tran Quang, Tuan Dang Thanh, Luc Van Meervelt

**Affiliations:** a Department of Chemistry, Hanoi National University of Education, 136 Xuan Thuy, Cau Giay, Hanoi, Vietnam; b Institute of Natural Sciences, Hanoi National University of Education, 136 Xuan Thuy, Cau Giay, Hanoi, Vietnam; c Institute of Chemistry, Vietnamese Academy of Science and Technology (VAST), 18 Hoang Quoc Viet, Hanoi, Vietnam; d Department of Chemistry, KU Leuven, Biomolecular Architecture, Celestijnenlaan 200F, Leuven (Heverlee), B-3001, Belgium; Vienna University of Technology, Austria

**Keywords:** crystal structure, Zn(II) complex, Cu(II) complex, anti­cancer activity

## Abstract

Two novel complexes, [Cu(**T4**)Cl_2_] and [Zn(**T4**)Cl_2_], were synthesized from 1,1′-[(3-fluoro­phen­yl)methyl­ene]bis­[3-(3-fluoro­phen­yl)imidazo[1,5-*a*]pyridine] (**T4**), and copper(II) and zinc(II) chloride, respectively. The structures of these complexes were confirmed using ESI-MS, IR and ^1^H NMR spectra. The results reveal mononuclear structures in which the central metal atoms are coordinated by two N atoms from the imidazole rings and two Cl ligands. The structure of the **CuT4** complex [systematic name: di­chlorido­{1,1′-[(3-fluoro­phen­yl)methyl­ene]bis­[3-(3-fluoro­phen­yl)imidazo[1,5-*a*]pyridine]-κ^2^*N*,*N*′}copper(II), [CuCl_2_(C_33_H_21_F_3_N_4_)], was confirmed by single-crystal X-ray diffraction. The Cu^II^ atom adopts a distorted tetra­hedral coordination environment with an N_2_Cl_2_ coordination set. The predominant features of the crystal packing are C—H⋯F, C—H⋯π and C—F⋯π inter­actions. Biological evaluations demonstrated that both complexes exhibit enhanced anti­cancer activity compared to the free ligand, with IC_50_ values ranging between 18.93 and 67.06 µ*M*. Notably, the Cu^II^ complex displays excellent inhibitory activity against the MCF7 breast cancer cell line (IC_50_ = 27.99 µ*M*), approximately twice as effective as cisplatin. Conversely, the **ZnT4** complex shows greater efficacy against Hep-G2 and A549 lung cancer cell lines, with IC_50_ values between 18.93 and 24.83 µ*M*. The results suggest that Cu^II^ and Zn^II^ complexes of **T4** show potential as cancer treatment agents.

## Abstract 2

The Cu^II^ and Zn^II^ complexes containing 1,1′-[(3-fluoro­phen­yl)methyl­ene]bis­[3-(3-fluoro­phen­yl)imidazo[1,5-*a*]pyridine] show potential as cancer treatment agents. The crystal-structure determination of the Cu^II^ complex confirms a distorted tetra­hedral N_2_Cl_2_ coordination set for the Cu^II^ atom.

## 1. Chemical context

Cancer remains a significant global health challenge, with high mortality rates worldwide. According to data from the World Health Organization (WHO), Vietnam ranks 90th out of 185 countries in terms of new cancer cases and 50th in cancer-related mortality (Globocan Vietnam, 2022 ▸). This creates an urgent need for the discovery of innovative and effective anti­cancer therapies. Platinum(II)-based complexes, such as cisplatin, carboplatin, and oxaliplatin, have been playing an important role in cancer chemotherapy for many cancer types worldwide. However, their clinical efficacy is often limited by significant side effects, including high toxicity, drug resistance and a lack of responsiveness across all cancer types (Johnstone *et al.*, 2016 ▸). Consequently, research efforts are increasingly focused on the discovery of novel compounds that exhibit improved anti­cancer activity and reduced toxicity profiles (Thi Hong Hai *et al.*, 2019 ▸; Thong *et al.*, 2022 ▸; Linh *et al.*, 2024 ▸). Besides the fact that the research into new Pt^II^ complexes is still ongoing despite high costs, recent efforts have also been made to investigate complexes of other *d*-block metals such as Cu^II^, Zn^II^, *etc*. These complexes offer potential advantages over platinum-based drugs, including lower cost and potentially reduced toxicity (Gou *et al.*, 2022 ▸; Agarwal *et al.*, 2022 ▸). Notably, several Cu^II^ and Zn^II^ complexes with heterocyclic N-containing polydentate ligands, such as quinoline and pyridine derivatives, have demonstrated promising anti­cancer activity, in some cases exceeding that of cisplatin (Hong *et al.*, 2022 ▸; Nguyen *et al.*, 2024 ▸; Rani & Roy, 2023 ▸).
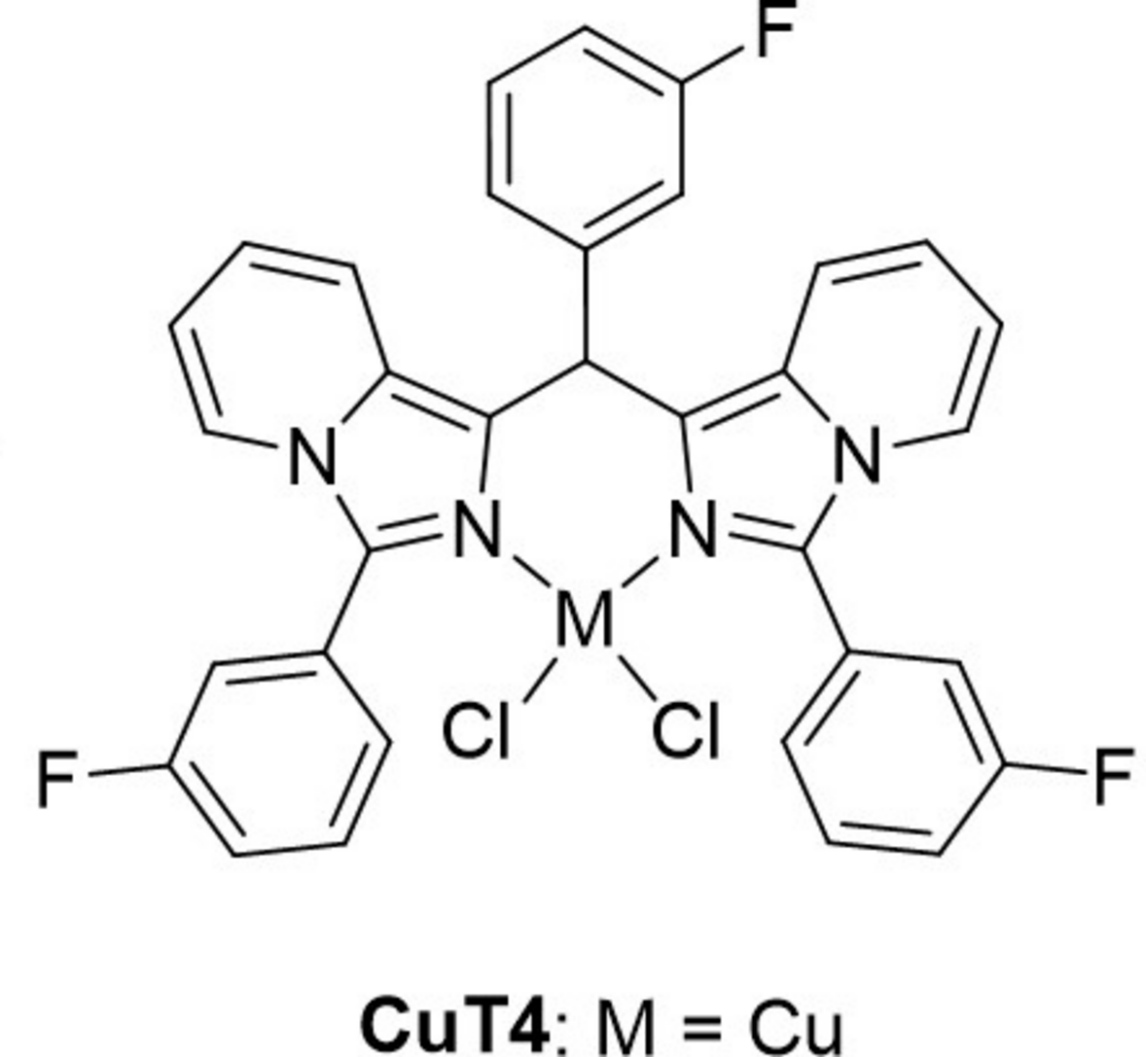


**Figure 1:**
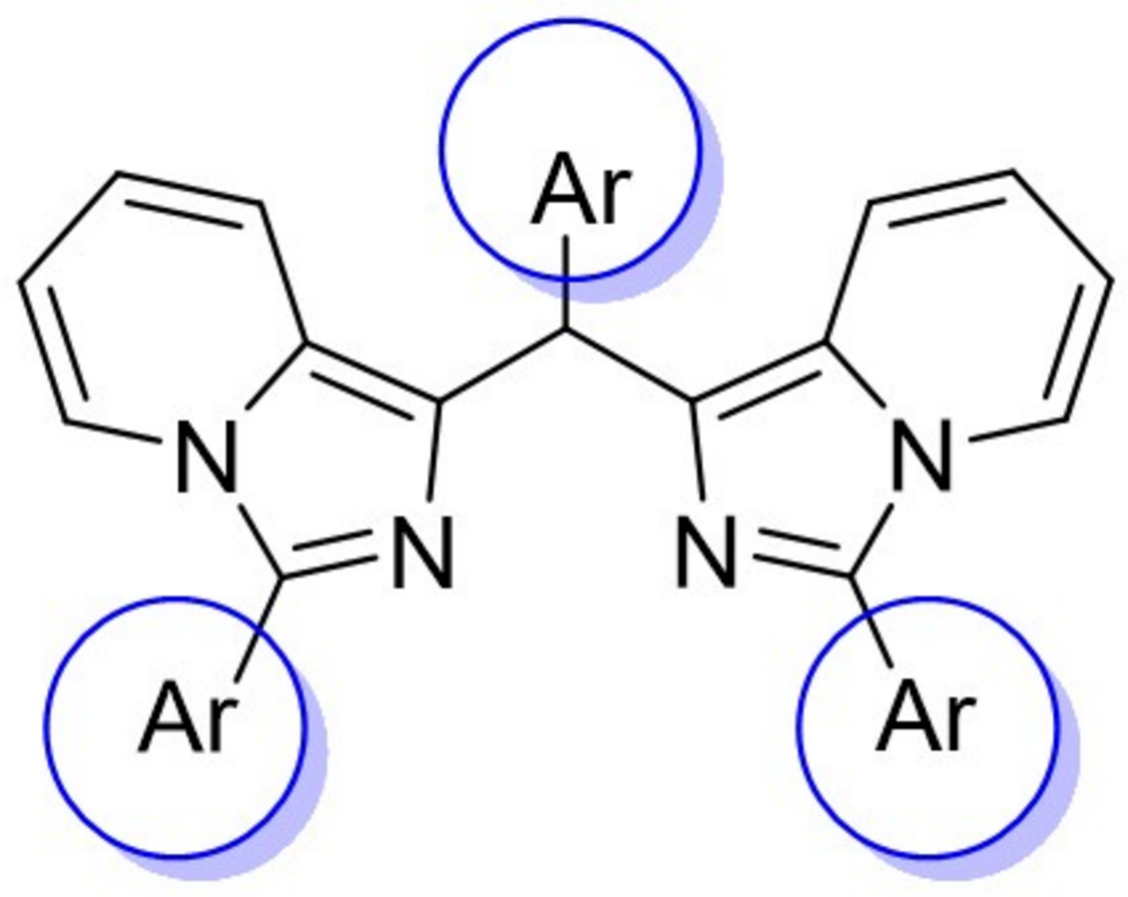
Chemical structure of imidazo[1,5-*a*]pyridine compounds (ImPys).

Among the various heterocyclic ligands explored for anti­cancer applications, imidazo[1,5-*a*]pyridines (ImPys) have emerged as a promising class of compounds (Fig. 1 ▸). Characterized by their aromatic ring systems and two heterocyclic nitro­gen atoms (Fig. 1 ▸), ImPys exhibit a diverse range of biological activities, including being anti­cancer agents (Priyanga *et al.*, 2019 ▸; Roy *et al.*, 2011 ▸), cardiotonic agents (Davey *et al.*, 1987 ▸), exhibiting anti-HIV activity (Kim *et al.*, 2005 ▸), having use in Alzheimer’s treatment (Nirogi *et al.*, 2015 ▸) and as anti-inflammatory agents (Fauber *et al.*, 2015 ▸). Furthermore, their extended π-conjugated systems make them attractive candidates for optoelectronic applications, as fluorescent sensors, and as cell-imaging markers (Volpi, 2022 ▸; Volpi *et al.*, 2017 ▸; Yagishita *et al.*, 2018 ▸). Additionally, the incorporation of ImPy cores into carbene ligands has led to intriguing applications in catalysis (Yagishita *et al.*, 2020 ▸).

The compound 1,1′-[(3-fluoro­phen­yl)methyl­ene]bis­[3-(3-fluoro­phen­yl)imidazo[1,5-*a*]pyridine] (**T4**) was synthesized according to a previously reported procedure (Phuc *et al.*, 2023 ▸). This ligand is capable of coordinating with transition-metal ions through two N atoms to form six-membered chelate ring complexes. The presence of multiple aromatic rings and fluorine substituents suggests that its complexes with *d*-block metal ions, such as Cu^II^ and Zn^II^, may exhibit cytotoxicity towards cancer cells. In the present study, two novel Cu^II^ and Zn^II^ complexes, [Cu(T4)Cl_2_] and [Zn(T4)Cl_2_], were synthesized. The structure of the **CuT4** complex was confirmed by single-crystal X-ray diffraction, while the structure of **ZnT4** complex was characterized by ^1^H NMR, ESI-MS and IR spectroscopy. Both complexes were found to be mononuclear, with the metal ion coordinated by the ligand *via* two nitro­gen atoms and completed with two chlorido ligands, resulting in a four-coordinate coordination environment. The cytotoxic activity of these complexes was evaluated against a panel of human cancer cell lines, including carcinoma cells (KB), liver cancer cells (Hep-G2), lung cancer cells (A549) and breast cancer cells (MCF7).

## 2. Structural commentary

**Figure 2:**
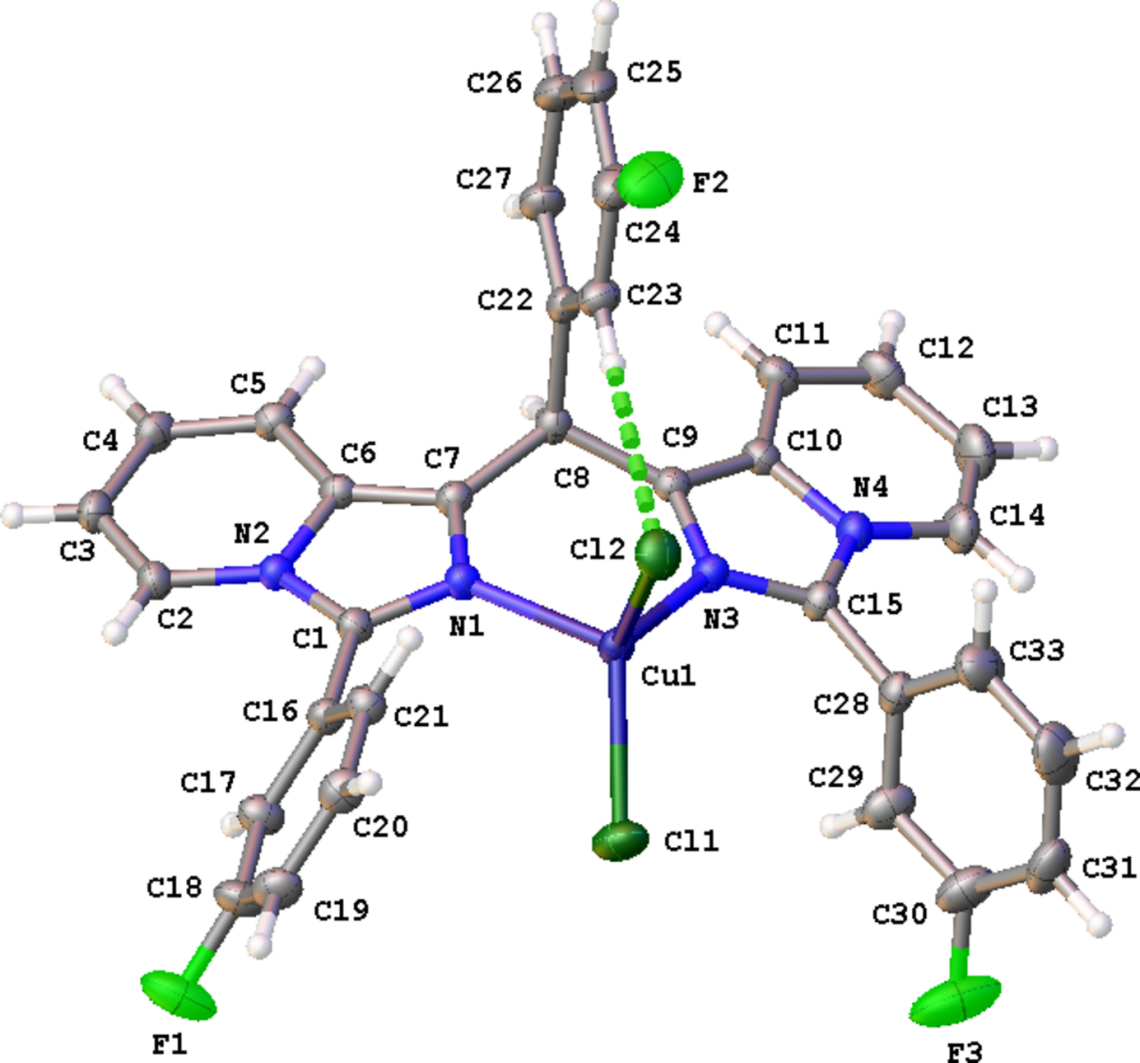
The mol­ecular structure of **CuT4** showing the atom-labeling scheme and displacement ellipsoids at the 30% probability level. The C—H⋯Cl inter­action is shown as a green dashed line.

The complex **CuT4** crystallizes in the triclinic space group *P*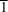 with one mol­ecule in the asymmetric unit (Fig. 2 ▸). The Cu^II^ atom displays a distorted tetra­hedral coordination with two N atoms from the imidazole rings and two Cl atoms. This is confirmed by the value of 0.90 for the τ_4_ parameter (Yang *et al.*, 2007 ▸). Typically, for Cu^II^ with coordination number 4, a square-planar coordination is observed. The tetra­hedral environment is most likely due to the steric constraints of the ligand and stabilized by the intra­molecular C23—H23⋯Cl2 inter­action (Table 1 ▸).

The boat conformation of the central Cu-containing six-membered ring is confirmed by the puckering parameters *Q* = 0.3947 (19) Å, θ = 91.2 (3) and φ = 352.3 (3)° (Cremer & Pople, 1975 ▸). The imidazo[1,5-*a*]pyridine rings make a dihedral angle of 26.76 (8)°. The mutual angle between the imidazo[1,5-*a*]pyridine ring containing N1, N2 and phenyl ring C16–C21 is 52.66 (11)°, lower than for the imidazo[1,5-*a*]pyridine ring containing N3, N4 and phenyl ring C28–C33, which is 85.22 (12)°, thus preventing the inter­nal mirror plane through atoms Cu1, Cl1, Cl2, C8 and C22–C27. The plane of phenyl ring C22–C27 makes an angle of 86.09 (11)° with the best least-squares plane through the central Cu-containing six-membered ring, allowing a short intra­molecular C23—H23⋯Cl2 contact (Table 1 ▸), as discussed above.

## 3. Supra­molecular features

**Figure 3:**
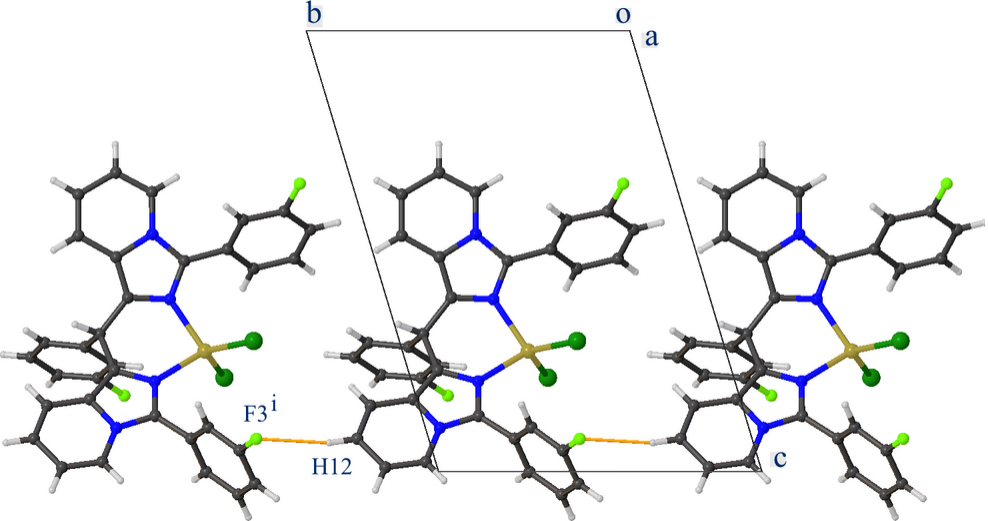
Partial crystal packing of **CuT4** viewed along the *a* axis showing the C—H⋯F inter­actions as orange lines. Further details are given in Table 1 ▸.

**Figure 4:**
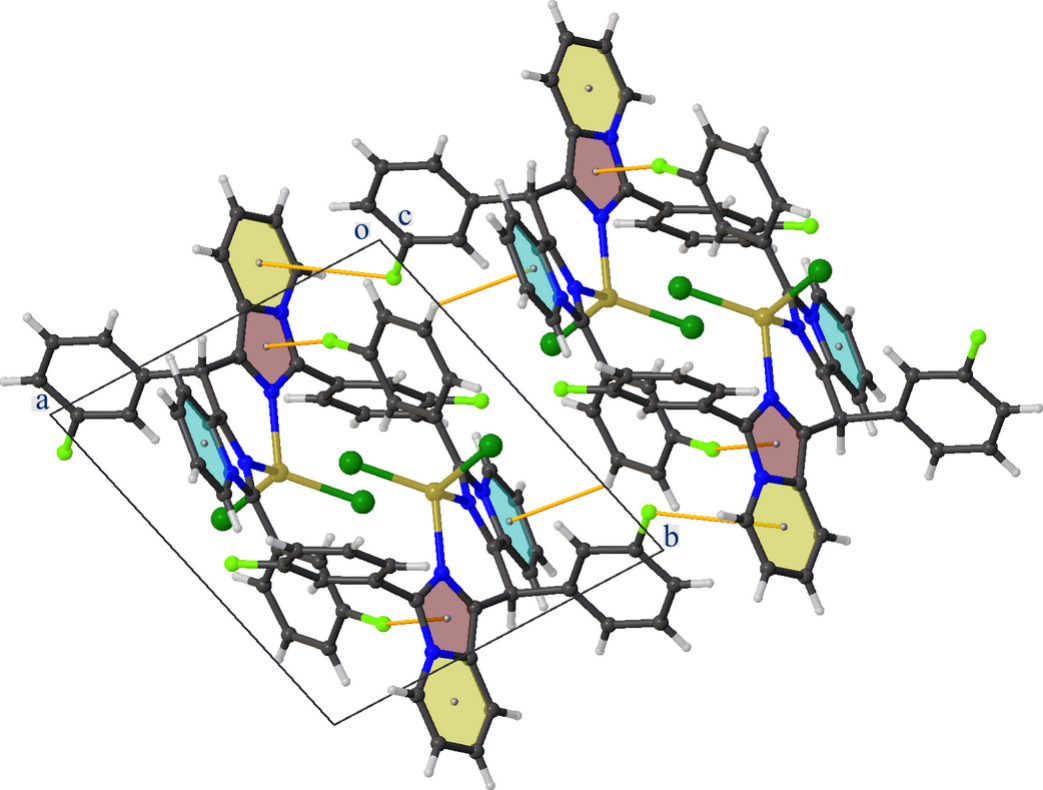
Partial crystal packing of **CuT4** viewed along the *c* axis showing C—H⋯π and C—F⋯π inter­actions as orange lines. Further details are given in Table 1 ▸.

The crystal packing of **CuT4** is built up by C—H⋯F, C—H⋯π and C—F⋯π inter­actions (Table 1 ▸). One of the pyridine H atoms (H12) forms a C—H⋯F hydrogen bond with atom F3 of an adjacent complex resulting in chain formation parallel to the *b-*axis direction (Fig. 3 ▸). A second chain parallel to the *a-*axis direction is created by a C—F⋯π inter­action between F2 and one of the pyridine rings (shown in yellow in Fig. 4 ▸). Furthermore, two types of inversion dimers are formed. The first one is the result of a C—H⋯π inter­action between H20 and one of the pyridine rings (N3/C2–C6, shown in blue in Fig. 4 ▸), and the second is a C—F⋯π inter­action between F1 and one of the imidazole rings (shown in brown in Fig. 4 ▸).

## 4. Database survey

A search of the Cambridge Structural Database (CSD, version 5.45, updated September 2024; Groom *et al.*, 2016 ▸) yielded 946 structures in which a Cu atom is coordinated by two N atoms forming part of a ring and two Cl ligands. We note that the CSD contains 72 entries containing the fragment bis­(imidazol-1-yl)methane, with 29 structures having a Cu atom bonded to both imidazole rings. In only eight of these hits does the Cu atom show a coordination number of four, but in none is the coordination sphere N_2_Cl_2_. The structure most related to **CuT4** is chlorido­{1,1′-[(pyridin-2-yl)methyl­ene]bis­[3-(pyridin-2-yl)imidazo[1,5-*a*]pyridine}­copper ethanol solvate (CSD refcode: ZEHHOL; Chen *et al.*, 2012 ▸), in which each 3-fluoro­phenyl group of **CuT4** is replaced by pyridine. In ZEHHOL, two N atoms of the two imidazo[1,5-*a*]pyridine units also coordinate to Cu^II^, but one of the two chloride ions is replaced by the N atom of the central pyridine ring with Cu. In **CuT4**, the central 3-fluoro­phenyl participates in a C—H⋯Cl inter­action. The bis­(imidazo[1,5-*a*]pyridin-1-yl)methane moiety is less planar in ZEHHOL [dihedral angle between the imidazo[1,5-*a*]pyridine rings = 119.58 (7)°] than in **CuT4**. The mol­ecular symmetry of ZEHHOL can be described by point group *C*_s_, with the central pyridine, Cu and Cl atoms lying in the mirror plane. Although this mol­ecular symmetry is also possible for **CuT4**, it is not observed here, which is probably related to the inter­molecular inter­actions described above.

## 5. Cytotoxic activity

**Figure 5:**
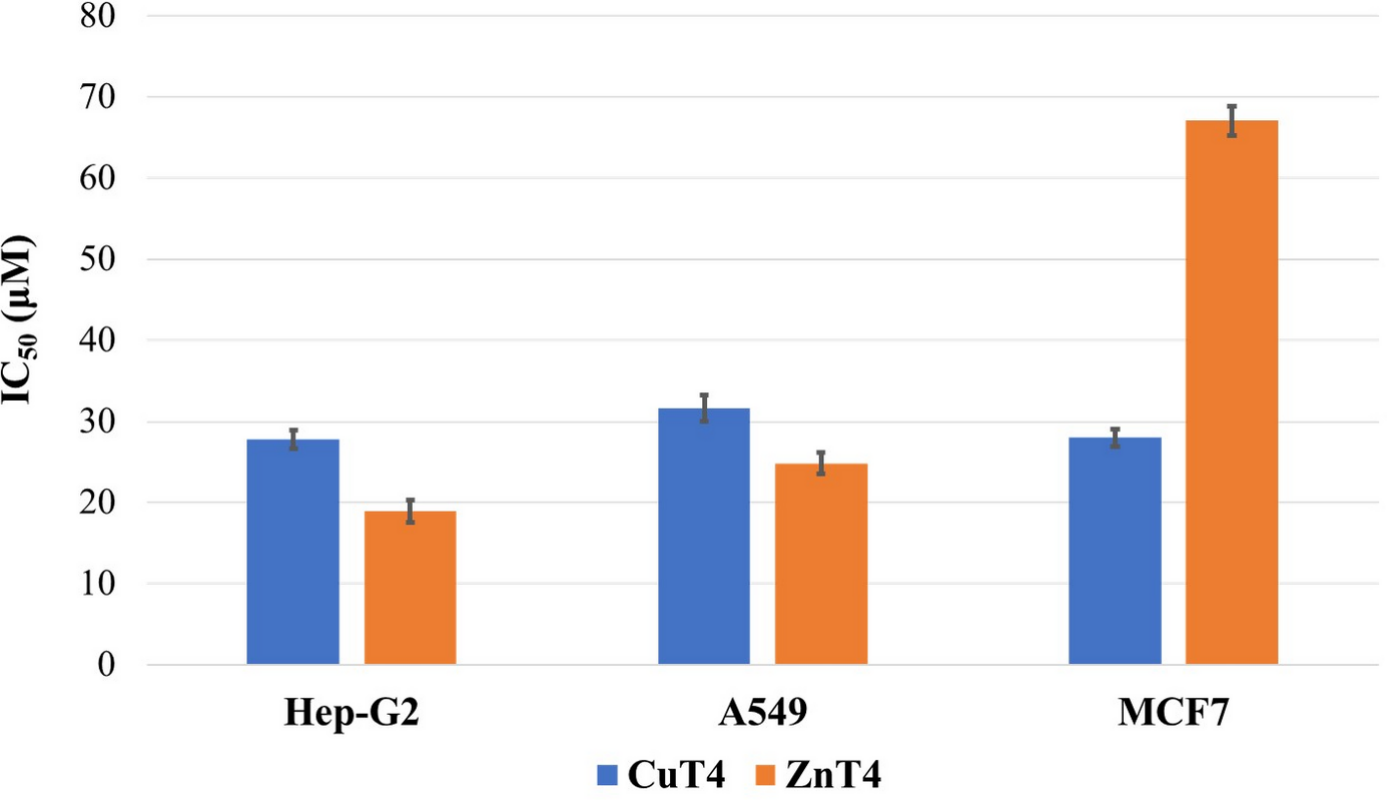
Cytotoxic activities for Hep-G2, A549 and MCF7 of **CuT4** and **ZnT4**.

The cytotoxicity activities of the ligand **T4** and its Cu^II^ and Zn^II^ complexes were evaluated against four human cancer cell lines, including KB (nasopharyngeal carcinoma), Hep-G2 (liver carcinoma), A549 (lung carcinoma), and MCF7 (breast adenocarcinoma). The colorimetric MTT assay was employed to assess cell viability following treatment with the compounds (Mosmann, 1983 ▸; Scudiero *et al.*, 1988 ▸; Malacrida *et al.*, 2019 ▸). The data presented in Fig. 5 ▸ and Table 2 ▸ indicate that both metal complexes exhibit better anti­cancer activity compared to the free ligand, with IC_50_ values ranging from approximately 18.93 to 67.06 µ*M*. Notably, the Cu^II^ complex **CuT4** demonstrates enhanced inhibitory activity against the MCF7 cell line, yielding an IC_50_ value of 27.99 µ*M*, approximately twice as effective as cisplatin, a widely used chemotherapeutic agent. Conversely, the **ZnT4** complex exhibits greater efficacy than **CuT4** in inhibiting cell growth in Hep-G2 and A549 cell lines, with IC_50_ values between 18.93 and 24.83 µ*M*.

## 6. Synthesis and crystallization

**Figure 6:**
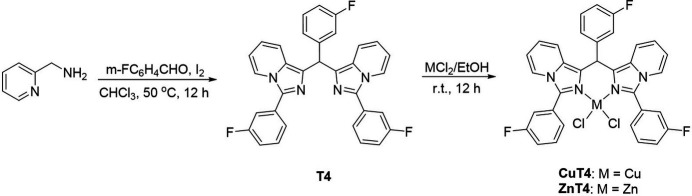
Synthesis scheme of **T4** and **CuT4, ZnT4** complexes.

The reaction sequence for **T4**, **CuT4** and **ZnT4** is shown in Fig. 6 ▸.

**Synthesis of 1,1′-[(3-fluoro­phen­yl)methyl­ene]bis­[3-(3-fluoro­phen­yl)imidazo[1,5-** ***a*** **]pyridine] (T4)**

The ligand **T4** was synthesized following the procedure reported by Phuc *et al.* (2023 ▸). ^1^H NMR (600 MHz, DMSO-*d_6_*, ppm): 6.37 (1H, *s*, CH), 6.55 (2H, *m*, Ar-H), 6.66 (2H, *m*, Ar-H), 6.94 (1H, *m*, Ar-H), 7.11–7.23 (3H, *m*, Ar-H), 7.29 (2H, *m*, Ar-H), 7.46–7.55 (4H, *m*, Ar-H), 7.60 (4H, *m*, Ar-H), 8.22 (2H, ^3^*J* = 7.2 Hz, ^4^*J* = 1.2 Hz, *dd*, Ar-H). IR (KBr, cm^−1^): 3071 (ν_C—H ar­yl_), 1613, 1585, 1516 (ν_C=C ar­yl_), 1470, 1444, 1407 (ν_C=N ar­yl_).

FT-IR and ^1^H NMR spectra of **T4** are given in the electronic supplementary information (ESI), Figs. S1 and S2, respectively.

**Synthesis of di­chlorido­{1,1′-[(3-fluoro­phen­yl)methyl­ene]bis­[3-(3-fluoro­phen­yl)imidazo[1,5-*****a*****]pyridine]-κ^2^*****N***,***N*****′}copper(II) (CuT4)**

To a solution of 1,1′-[(3-fluoro­phen­yl)methyl­ene]bis­[3-(3-fluoro­phen­yl)imidazo[1,5-*a*]pyridine] (53.26 mg, 0.10 mmol) completely dissolved in 3 ml of ethanol, a solution containing copper(II) chloride dihydrate (20.52 mg, 0.12 mmol) in 2 ml of ethanol was slowly added, resulting in the rapid formation of a green precipitate. After 12 h of continuous stirring at room temperature, the product **CuT4** was filtered and washed with cold ethanol (yield 89%). The **CuT4** complex exhibited poor solubility in ethanol but good solubility in DCM and DMSO. Recrystallization was performed at room temperature using vapor diffusion of *n*-hexane into a saturated solution of **CuT4** in a DCM–ethanol mixture (*v*/*v* = 1:1) yielding green single crystals after 24 h. IR (KBr, cm^−1^): 2964, 2886 (ν_C—H ar­yl_), 1560, 1523 (ν_C=C ar­yl_), 1433, 1405 (ν_C=N ar­yl_). MS (ESI+): 681.0 (100%), [Cu(**T4**)Cl_2_ + H_2_O + H]^+^, 744.9 (60%), [Cu(**T4**)Cl_2_ + DMSO + H]^+^, MS (ESI-): 631.5 (100%), [Cu(**T4**)Cl_2_ – Cl – 2H]^−^.

ESI-MS and FT-IR spectra of **CuT4** are given in the ESI, Figs. S3, S4 and S5, respectively. Fig. S6 shows a picture of the **CuT4** crystals.

**Synthesis of di­chlorido­{1,1′-[(3-fluoro­phen­yl)methyl­ene]bis­[3-(3-fluoro­phen­yl)imidazo[1,5-*****a*****]pyridine]-κ^2^*****N***,***N*****′}zinc(II) (ZnT4)**

To a solution of 1,1′-[(3-fluoro­phen­yl)methyl­ene]bis­[3-(3-fluoro­phen­yl)imidazo[1,5-*a*]pyridine] (53.26 mg, 0.10 mmol) completely dissolved in 3 ml of ethanol, a solution containing zinc(II) chloride (16.32 mg, 0.12 mmol) in 2 ml ethanol was slowly added, resulting in the rapid formation of a colorless precipitate. After 12 h of continuous stirring at room temperature, the product **ZnT4** was filtered and washed with cold ethanol (yield 85%). Recrystallization of **ZnT4** was performed as described for **CuT4**. ^1^H NMR (600 MHz, DMSO-*d_6_*, ppm): 6.374 (1H, *s*, CH), 6.713 (2H, ^3^*J* = 7.2 Hz, ^4^*J* = 1.2 Hz, td, Ar-H), 6.805 (2H, ^3^*J* = 7.2 Hz, t, Ar-H), 7.032 (1H, *m*, Ar-H), 7.259 7.350 (5H, *m*, Ar-H), 7.557 7.616 (4H, *m*, Ar-H), 7.660 7.677 (4H, *m*, Ar-H), 8.459 (2H, ^3^*J* = 7.2 Hz, d, Ar-H). IR (KBr, cm^−1^): 1618, 1608, 1585 (ν_C=C ar­yl_), 1525, 1471 (ν_C=N ar­yl_). MS (ESI+): 629.0 (60%), [Zn(**T4**)Cl_2_ – Cl]^+^.

ESI-MS, FT-IR and ^1^H NMR spectra of **ZnT4** are given in the ESI, Figs. S7, S8 and S9, respectively.

**Figure 7:**
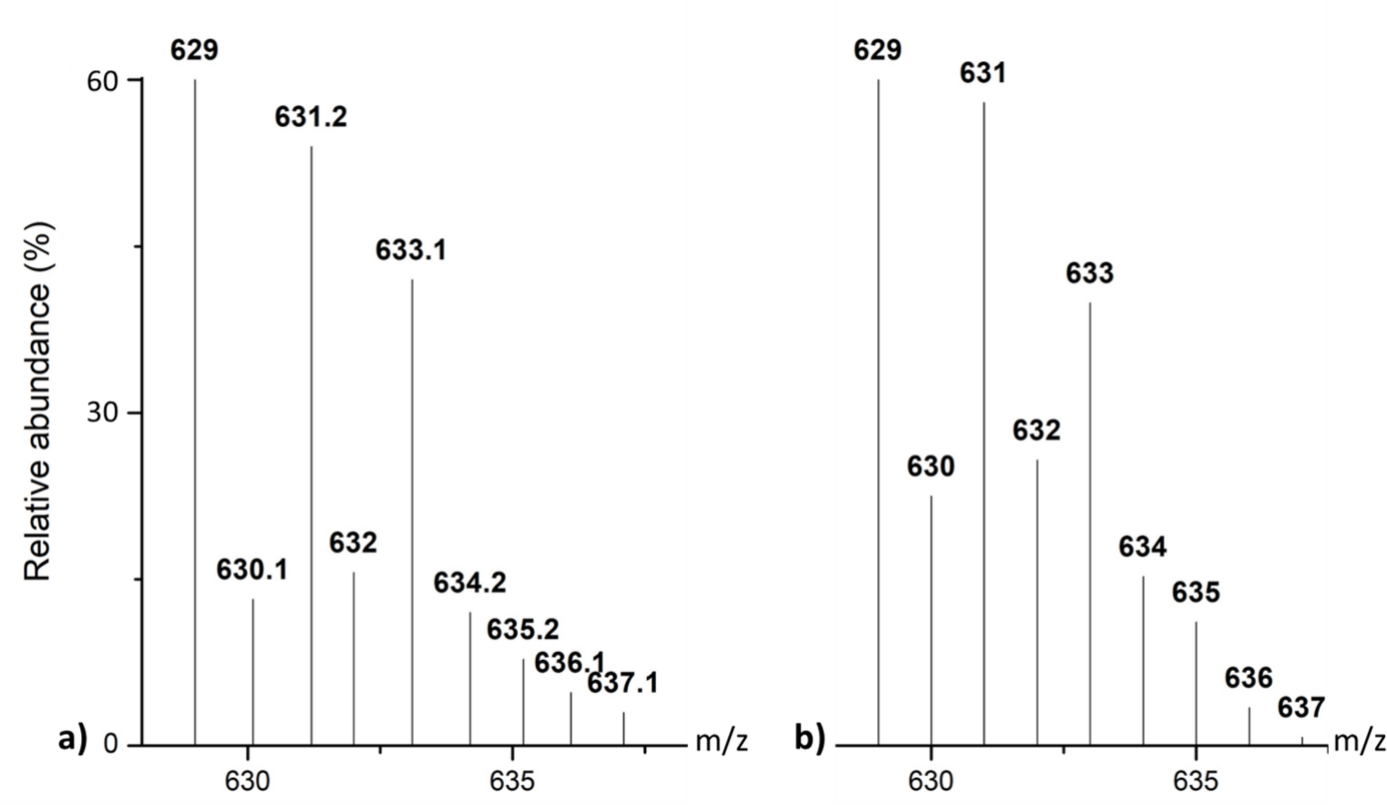
(*a*) Experimental isotope peaks and (*b*) theoretical isotope peaks of the fragment [Zn(**T4**)Cl_2_ – Cl]^+^ in the ESI+ spectrum of **ZnT4**.

In the positive-mode electrospray ionization mass spectrometry (ESI-MS) spectrum of the **ZnT4** complex, a peak cluster corresponding to the [Zn(**T4**)Cl]^+^ cation fragment was observed with a relative abundance of 60%, indicating that this fragment resulted from the release of one chlorido ligand from **ZnT4**. Furthermore, there was consistency between the experimental and calculated spectrum in the number and ratio of peaks in the [Zn(**T4**)Cl_2_ – Cl]^+^ ion peak cluster (Fig. 7 ▸). The IR spectrum of **ZnT4** further confirmed an increase in frequency of valence vibrations of aromatic C=N and C=C bonds. In addition, the ^1^H NMR spectrum of **ZnT4** revealed that all proton signals corresponding to the **T4** ligand were present, and that signals from all protons located near the coordination center are shifted compared to the free ligand. These spectroscopic data support the proposed mol­ecular formula for the **ZnT4** complex as [Zn(**T4**)Cl_2_], where the central Zn^II^ ion is coordinated by two nitro­gen atoms of the imidazole rings of the **T4** ligand while also binding to two chlorido ligands.

## 7. Refinement

Crystal data, data collection and structure refinement details are summarized in Table 3 ▸. All H atoms were placed in idealized positions and refined in riding mode with C—H distances of 0.93 (aromatic) and 0.98 Å (CH). Non-hydrogen atoms were refined anisotropically (rigid bond constraints for C24 and F2) and hydrogen atoms with isotropic temperature factors fixed at 1.2 times *U*_eq_ of the parent atoms.

## Supplementary Material

Crystal structure: contains datablock(s) I. DOI: 10.1107/S2056989024011617/wm5742sup1.cif

Structure factors: contains datablock(s) I. DOI: 10.1107/S2056989024011617/wm5742Isup2.hkl

Spectra for T4, CuT4 and ZnT4. DOI: 10.1107/S2056989024011617/wm5742sup3.docx

CCDC reference: 2406199

Additional supporting information:  crystallographic information; 3D view; checkCIF report

## Figures and Tables

**Table 1:** Hydrogen-bond geometry (Å, °) *Cg*1, *Cg*2 and *Cg*3 are the centroids of rings N2/C2–C6, N3/N4/C9–C10/C15 and N4/C10–C14, respectively.

*D*—H⋯*A*	*D*—H	H⋯*A*	*D*⋯*A*	*D*—H⋯*A*
C12—H12⋯F3^i^	0.93	2.47	3.384 (4)	166
C23—H23⋯Cl2	0.93	2.81	3.736 (3)	174
C20—H20⋯*Cg*1^ii^	0.93	2.77	3.477 (3)	133
C18—F1⋯*Cg*2^iii^	1.35 (1)	3.23 (1)	4.484 (3)	154 (1)
C24—F2⋯*Cg*3^iv^	1.36 (1)	3.57 (1)	4.284 (3)	113 (1)

Symmetry codes: (i) 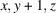; (ii) 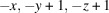; (iii) 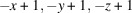; (iv) 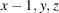.

**Table 2:** *In vitro* cytotoxicity of **CuT4**, **ZnT4** and reference compounds against four human cancer cell lines, IC_50_ in μ*M*

Compound	KB	Hep-G2	A549	MCF7
**CuT4**	34.18 ± 1.12	27.80 ± 1.14	31.63 ± 1.66	27.99 ± 1.11
**ZnT4**	-	18.93 ± 1.41	24.83 ± 1.30	67.06 ± 1.81
**T4**	> 100	> 100	> 100	> 100
Cisplatin^*a*^	15.3	13.3	-	45.7
Ellipticine	1.75 ± 0.08	1.71 ± 0.08	1.75 ± 0.08	1.71 ± 0.12

Note: (*a*) Chi *et al.* (2020 ▸).

**Table 3:** Experimental details

Crystal data	
Chemical formula	[CuCl_2_(C_33_H_21_F_3_N_4_)]
*M*_r_	664.98
Crystal system, space group	Triclinic, *P* 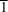
Temperature (K)	294
*a*, *b*, *c* (Å)	9.3994 (2), 10.8016 (3), 15.2355 (4)
α, β, γ (°)	104.272 (2), 99.186 (2), 101.529 (2)
*V* (Å^3^)	1432.87 (7)
*Z*	2
Radiation type	Mo *K*α
μ (mm^−1^)	1.00
Crystal size (mm)	0.3 × 0.3 × 0.1

Data collection	
Diffractometer	SuperNova, Single source at offset/far, Eos
Absorption correction	Multi-scan (*CrysAlis PRO*; Rigaku OD, 2023 ▸)
*T*_min_, *T*_max_	0.742, 1.000
No. of measured, independent and observed [*I* > 2σ(*I*)] reflections	29600, 5864, 4963
*R*_int_	0.033
(sin θ/λ)_max_ (Å^−1^)	0.625

Refinement	
*R*[*F*^2^ > 2σ(*F*^2^)], *wR*(*F*^2^), *S*	0.035, 0.089, 1.07
No. of reflections	5864
No. of parameters	388
No. of restraints	1
H-atom treatment	H-atom parameters constrained
Δρ_max_, Δρ_min_ (e Å^−3^)	0.73, −0.42

Computer programs: *CrysAlis PRO* (Rigaku OD, 2023 ▸), *SHELXT* (Sheldrick, 2015*a* ▸), *SHELXL* (Sheldrick, 2015*b* ▸) and *OLEX2* (Dolomanov *et al.*, 2009 ▸).

## supplementary crystallographic information

### Dichlorido{1,1'-[(3-fluorophenyl)methylene]bis[3-(3-fluorophenyl)imidazo[1,5-a]pyridine]-κ2N,N'}copper(II) Crystal data

**Table:**

[CuCl_2_(C_33_H_21_F_3_N_4_)]	*Z* = 2
*M**_r_* = 664.98	*F*(000) = 674
Triclinic, *P*1	*D*_x_ = 1.541 Mg m^−^^3^
*a* = 9.3994 (2) Å	Mo *K*α radiation, λ = 0.71073 Å
*b* = 10.8016 (3) Å	Cell parameters from 12271 reflections
*c* = 15.2355 (4) Å	θ = 2.6–26.4°
α = 104.272 (2)°	µ = 1.00 mm^−^^1^
β = 99.186 (2)°	*T* = 294 K
γ = 101.529 (2)°	Plate, green
*V* = 1432.87 (7) Å^3^	0.3 × 0.3 × 0.1 mm

### Dichlorido{1,1'-[(3-fluorophenyl)methylene]bis[3-(3-fluorophenyl)imidazo[1,5-a]pyridine]-κ2N,N'}copper(II) Data collection

**Table:**

SuperNova, Single source at offset/far, Eos diffractometer	5864 independent reflections
Radiation source: micro-focus sealed X-ray tube, SuperNova (Mo) X-ray Source	4963 reflections with *I* > 2σ(*I*)
Mirror monochromator	*R*_int_ = 0.033
Detector resolution: 15.9631 pixels mm^-1^	θ_max_ = 26.4°, θ_min_ = 2.4°
ω scans	*h* = −11→11
Absorption correction: multi-scan (CrysAlisPro; Rigaku OD, 2023)	*k* = −13→13
*T*_min_ = 0.742, *T*_max_ = 1.000	*l* = −19→19
29600 measured reflections	

### Dichlorido{1,1'-[(3-fluorophenyl)methylene]bis[3-(3-fluorophenyl)imidazo[1,5-a]pyridine]-κ2N,N'}copper(II) Refinement

**Table:**

Refinement on *F*^2^	Primary atom site location: dual
Least-squares matrix: full	Hydrogen site location: inferred from neighbouring sites
*R*[*F*^2^ > 2σ(*F*^2^)] = 0.035	H-atom parameters constrained
*wR*(*F*^2^) = 0.089	*w* = 1/[σ^2^(*F*_o_^2^) + (0.0333*P*)^2^ + 0.9126*P*] where *P* = (*F*_o_^2^ + 2*F*_c_^2^)/3
*S* = 1.07	(Δ/σ)_max_ = 0.001
5864 reflections	Δρ_max_ = 0.73 e Å^−^^3^
388 parameters	Δρ_min_ = −0.42 e Å^−^^3^
1 restraint	

### Dichlorido{1,1'-[(3-fluorophenyl)methylene]bis[3-(3-fluorophenyl)imidazo[1,5-a]pyridine]-κ2N,N'}copper(II) Special details

**Table:**

Geometry. All esds (except the esd in the dihedral angle between two l.s. planes) are estimated using the full covariance matrix. The cell esds are taken into account individually in the estimation of esds in distances, angles and torsion angles; correlations between esds in cell parameters are only used when they are defined by crystal symmetry. An approximate (isotropic) treatment of cell esds is used for estimating esds involving l.s. planes.

### Dichlorido{1,1'-[(3-fluorophenyl)methylene]bis[3-(3-fluorophenyl)imidazo[1,5-a]pyridine]-κ2N,N'}copper(II) Fractional atomic coordinates and isotropic or equivalent isotropic displacement parameters (Å^2^)

**Table:**

	*x*	*y*	*z*	*U*_iso_*/*U*_eq_	
Cu1	0.35886 (3)	0.61095 (3)	0.72622 (2)	0.03217 (9)	
Cl1	0.47558 (8)	0.45051 (7)	0.70330 (6)	0.05887 (19)	
F1	0.2895 (2)	0.16205 (17)	0.34675 (15)	0.0867 (6)	
N1	0.3092 (2)	0.67074 (18)	0.60887 (12)	0.0326 (4)	
C1	0.2618 (2)	0.5909 (2)	0.52193 (15)	0.0321 (5)	
Cl2	0.15785 (7)	0.57489 (7)	0.78641 (5)	0.05071 (17)	
F2	−0.0417 (2)	0.9011 (2)	0.82735 (14)	0.0835 (6)	
N2	0.26041 (19)	0.66471 (17)	0.46111 (12)	0.0309 (4)	
C2	0.2068 (3)	0.6273 (2)	0.36579 (15)	0.0363 (5)	
H2	0.170531	0.538609	0.332765	0.044*	
F3	0.9069 (3)	0.5333 (2)	0.9251 (2)	0.1161 (9)	
N3	0.4955 (2)	0.79439 (18)	0.79499 (13)	0.0349 (4)	
C3	0.2079 (3)	0.7207 (2)	0.32181 (16)	0.0406 (5)	
H3	0.170817	0.696206	0.257997	0.049*	
N4	0.6629 (2)	0.95568 (19)	0.90043 (13)	0.0369 (4)	
C4	0.2652 (3)	0.8570 (2)	0.37145 (16)	0.0400 (5)	
H4	0.268768	0.920211	0.339433	0.048*	
C5	0.3143 (2)	0.8951 (2)	0.46476 (16)	0.0351 (5)	
H5	0.350175	0.984130	0.497013	0.042*	
C6	0.3107 (2)	0.7979 (2)	0.51320 (15)	0.0304 (5)	
C7	0.3401 (2)	0.7989 (2)	0.60494 (15)	0.0310 (5)	
C8	0.3920 (2)	0.9203 (2)	0.68733 (15)	0.0331 (5)	
H8	0.451210	0.987882	0.665955	0.040*	
C9	0.4942 (2)	0.9085 (2)	0.77024 (15)	0.0321 (5)	
C10	0.5982 (2)	1.0114 (2)	0.83562 (15)	0.0345 (5)	
C11	0.6495 (3)	1.1494 (2)	0.85004 (17)	0.0422 (6)	
H11	0.608484	1.189958	0.808583	0.051*	
C12	0.7586 (3)	1.2208 (3)	0.92489 (19)	0.0545 (7)	
H12	0.793561	1.310941	0.934285	0.065*	
C13	0.8212 (3)	1.1605 (3)	0.9897 (2)	0.0597 (8)	
H13	0.895268	1.212013	1.041211	0.072*	
C14	0.7747 (3)	1.0308 (3)	0.97746 (18)	0.0511 (7)	
H14	0.816306	0.991523	1.019597	0.061*	
C15	0.5985 (2)	0.8244 (2)	0.87332 (16)	0.0366 (5)	
C16	0.2168 (2)	0.4460 (2)	0.49540 (15)	0.0337 (5)	
C17	0.2754 (3)	0.3710 (2)	0.42919 (17)	0.0433 (6)	
H17	0.342033	0.410741	0.399214	0.052*	
C18	0.2314 (3)	0.2358 (3)	0.40951 (19)	0.0506 (7)	
C19	0.1331 (3)	0.1725 (3)	0.4508 (2)	0.0499 (6)	
H19	0.104923	0.080823	0.434727	0.060*	
C20	0.0768 (3)	0.2477 (2)	0.51667 (18)	0.0455 (6)	
H20	0.010776	0.206716	0.546413	0.055*	
C21	0.1176 (3)	0.3841 (2)	0.53914 (16)	0.0384 (5)	
H21	0.078456	0.434347	0.583540	0.046*	
C22	0.2634 (3)	0.9763 (2)	0.71679 (15)	0.0353 (5)	
C23	0.1630 (3)	0.9087 (3)	0.75644 (17)	0.0440 (6)	
H23	0.167992	0.825824	0.762208	0.053*	
C24	0.0557 (3)	0.9667 (3)	0.78711 (19)	0.0535 (7)	
C25	0.0420 (3)	1.0879 (3)	0.7803 (2)	0.0594 (8)	
H25	−0.031877	1.124201	0.802104	0.071*	
C26	0.1412 (4)	1.1541 (3)	0.7400 (2)	0.0599 (8)	
H26	0.134416	1.236611	0.734309	0.072*	
C27	0.2515 (3)	1.0991 (2)	0.70780 (18)	0.0470 (6)	
H27	0.317434	1.144510	0.680199	0.056*	
C28	0.6448 (3)	0.7346 (2)	0.92467 (16)	0.0390 (5)	
C29	0.7529 (3)	0.6719 (3)	0.8994 (2)	0.0534 (7)	
H29	0.791538	0.681133	0.848171	0.064*	
C30	0.8018 (3)	0.5958 (3)	0.9516 (2)	0.0614 (8)	
C31	0.7501 (4)	0.5788 (3)	1.0267 (2)	0.0659 (9)	
H31	0.786550	0.526765	1.060648	0.079*	
C32	0.6423 (5)	0.6407 (4)	1.0515 (2)	0.0765 (10)	
H32	0.604302	0.630202	1.102705	0.092*	
C33	0.5898 (4)	0.7190 (3)	1.0003 (2)	0.0651 (8)	
H33	0.516962	0.760891	1.017591	0.078*	

### Dichlorido{1,1'-[(3-fluorophenyl)methylene]bis[3-(3-fluorophenyl)imidazo[1,5-a]pyridine]-κ2N,N'}copper(II) Atomic displacement parameters (Å^2^)

**Table:**

	*U*^11^	*U*^22^	*U*^33^	*U*^12^	*U*^13^	*U*^23^
Cu1	0.03619 (16)	0.02714 (15)	0.03347 (15)	0.00529 (11)	0.00489 (11)	0.01311 (11)
Cl1	0.0579 (4)	0.0430 (4)	0.0797 (5)	0.0217 (3)	0.0113 (4)	0.0201 (3)
F1	0.1089 (16)	0.0487 (10)	0.1097 (16)	0.0250 (10)	0.0649 (13)	0.0058 (10)
N1	0.0380 (10)	0.0286 (9)	0.0321 (10)	0.0075 (8)	0.0080 (8)	0.0108 (8)
C1	0.0347 (11)	0.0311 (11)	0.0318 (11)	0.0080 (9)	0.0090 (9)	0.0102 (9)
Cl2	0.0516 (4)	0.0562 (4)	0.0474 (4)	0.0068 (3)	0.0177 (3)	0.0216 (3)
F2	0.0684 (12)	0.1194 (17)	0.0961 (14)	0.0426 (11)	0.0481 (10)	0.0569 (13)
N2	0.0336 (9)	0.0290 (9)	0.0311 (9)	0.0078 (8)	0.0079 (7)	0.0101 (8)
C2	0.0413 (13)	0.0366 (12)	0.0297 (11)	0.0097 (10)	0.0074 (9)	0.0077 (9)
F3	0.0980 (17)	0.1124 (19)	0.198 (3)	0.0665 (15)	0.0602 (17)	0.1031 (19)
N3	0.0350 (10)	0.0329 (10)	0.0357 (10)	0.0077 (8)	0.0038 (8)	0.0113 (8)
C3	0.0468 (14)	0.0467 (14)	0.0309 (12)	0.0141 (11)	0.0078 (10)	0.0140 (10)
N4	0.0372 (10)	0.0361 (11)	0.0332 (10)	0.0065 (8)	0.0038 (8)	0.0069 (8)
C4	0.0444 (13)	0.0413 (13)	0.0410 (13)	0.0117 (11)	0.0112 (10)	0.0219 (11)
C5	0.0336 (12)	0.0310 (12)	0.0411 (13)	0.0056 (9)	0.0072 (10)	0.0137 (10)
C6	0.0295 (11)	0.0268 (11)	0.0345 (11)	0.0061 (8)	0.0069 (9)	0.0085 (9)
C7	0.0313 (11)	0.0282 (11)	0.0338 (11)	0.0067 (9)	0.0064 (9)	0.0107 (9)
C8	0.0365 (12)	0.0285 (11)	0.0332 (11)	0.0052 (9)	0.0051 (9)	0.0105 (9)
C9	0.0327 (11)	0.0300 (11)	0.0337 (11)	0.0081 (9)	0.0081 (9)	0.0086 (9)
C10	0.0362 (12)	0.0332 (12)	0.0339 (12)	0.0093 (9)	0.0105 (9)	0.0070 (9)
C11	0.0507 (14)	0.0333 (13)	0.0413 (13)	0.0093 (11)	0.0142 (11)	0.0070 (10)
C12	0.0626 (18)	0.0336 (14)	0.0540 (17)	−0.0005 (12)	0.0105 (13)	0.0003 (12)
C13	0.0593 (18)	0.0528 (18)	0.0449 (16)	−0.0036 (14)	−0.0041 (13)	−0.0017 (13)
C14	0.0505 (15)	0.0503 (16)	0.0390 (14)	0.0042 (12)	−0.0045 (11)	0.0045 (12)
C15	0.0343 (12)	0.0378 (13)	0.0350 (12)	0.0063 (10)	0.0026 (9)	0.0107 (10)
C16	0.0387 (12)	0.0275 (11)	0.0342 (12)	0.0077 (9)	0.0058 (9)	0.0093 (9)
C17	0.0498 (14)	0.0358 (13)	0.0478 (14)	0.0108 (11)	0.0187 (12)	0.0134 (11)
C18	0.0603 (17)	0.0336 (13)	0.0574 (16)	0.0180 (12)	0.0185 (13)	0.0039 (12)
C19	0.0530 (16)	0.0298 (13)	0.0652 (18)	0.0076 (11)	0.0089 (13)	0.0153 (12)
C20	0.0421 (14)	0.0389 (14)	0.0551 (16)	0.0030 (11)	0.0088 (12)	0.0198 (12)
C21	0.0405 (13)	0.0351 (12)	0.0387 (13)	0.0078 (10)	0.0098 (10)	0.0096 (10)
C22	0.0410 (12)	0.0333 (12)	0.0290 (11)	0.0112 (10)	0.0007 (9)	0.0073 (9)
C23	0.0458 (14)	0.0457 (15)	0.0463 (14)	0.0184 (12)	0.0116 (11)	0.0174 (12)
C24	0.0493 (15)	0.073 (2)	0.0460 (15)	0.0243 (13)	0.0129 (11)	0.0218 (13)
C25	0.0601 (18)	0.076 (2)	0.0488 (16)	0.0425 (16)	0.0098 (14)	0.0115 (15)
C26	0.073 (2)	0.0493 (16)	0.0602 (18)	0.0351 (15)	0.0036 (15)	0.0121 (14)
C27	0.0573 (16)	0.0369 (13)	0.0498 (15)	0.0180 (12)	0.0090 (12)	0.0145 (11)
C28	0.0391 (13)	0.0387 (13)	0.0344 (12)	0.0050 (10)	−0.0012 (10)	0.0115 (10)
C29	0.0510 (16)	0.0518 (16)	0.0690 (19)	0.0140 (13)	0.0183 (14)	0.0339 (14)
C30	0.0516 (17)	0.0530 (17)	0.089 (2)	0.0164 (14)	0.0101 (16)	0.0390 (17)
C31	0.081 (2)	0.0512 (18)	0.0596 (19)	0.0098 (16)	−0.0113 (16)	0.0280 (15)
C32	0.119 (3)	0.072 (2)	0.0448 (17)	0.017 (2)	0.0236 (18)	0.0300 (16)
C33	0.087 (2)	0.071 (2)	0.0524 (17)	0.0322 (18)	0.0300 (16)	0.0253 (16)

### Dichlorido{1,1'-[(3-fluorophenyl)methylene]bis[3-(3-fluorophenyl)imidazo[1,5-a]pyridine]-κ2N,N'}copper(II) Geometric parameters (Å, º)

**Table:**

Cu1—Cl1	2.2164 (7)	C12—C13	1.425 (4)
Cu1—N1	2.0626 (18)	C13—H13	0.9300
Cu1—Cl2	2.2356 (7)	C13—C14	1.338 (4)
Cu1—N3	2.0503 (19)	C14—H14	0.9300
F1—C18	1.346 (3)	C15—C28	1.476 (3)
N1—C1	1.338 (3)	C16—C17	1.389 (3)
N1—C7	1.375 (3)	C16—C21	1.389 (3)
C1—N2	1.363 (3)	C17—H17	0.9300
C1—C16	1.470 (3)	C17—C18	1.378 (3)
F2—C24	1.360 (3)	C18—C19	1.365 (4)
N2—C2	1.386 (3)	C19—H19	0.9300
N2—C6	1.403 (3)	C19—C20	1.374 (4)
C2—H2	0.9300	C20—H20	0.9300
C2—C3	1.341 (3)	C20—C21	1.386 (3)
F3—C30	1.362 (4)	C21—H21	0.9300
N3—C9	1.376 (3)	C22—C23	1.383 (3)
N3—C15	1.334 (3)	C22—C27	1.391 (3)
C3—H3	0.9300	C23—H23	0.9300
C3—C4	1.426 (3)	C23—C24	1.374 (4)
N4—C10	1.398 (3)	C24—C25	1.368 (4)
N4—C14	1.391 (3)	C25—H25	0.9300
N4—C15	1.355 (3)	C25—C26	1.374 (4)
C4—H4	0.9300	C26—H26	0.9300
C4—C5	1.353 (3)	C26—C27	1.391 (4)
C5—H5	0.9300	C27—H27	0.9300
C5—C6	1.422 (3)	C28—C29	1.382 (4)
C6—C7	1.378 (3)	C28—C33	1.370 (4)
C7—C8	1.507 (3)	C29—H29	0.9300
C8—H8	0.9800	C29—C30	1.370 (4)
C8—C9	1.508 (3)	C30—C31	1.350 (5)
C8—C22	1.536 (3)	C31—H31	0.9300
C9—C10	1.376 (3)	C31—C32	1.372 (5)
C10—C11	1.421 (3)	C32—H32	0.9300
C11—H11	0.9300	C32—C33	1.392 (4)
C11—C12	1.351 (4)	C33—H33	0.9300
C12—H12	0.9300		
			
Cl1—Cu1—Cl2	116.29 (3)	N4—C14—H14	120.7
N1—Cu1—Cl1	112.26 (6)	C13—C14—N4	118.7 (3)
N1—Cu1—Cl2	110.16 (6)	C13—C14—H14	120.7
N3—Cu1—Cl1	114.35 (6)	N3—C15—N4	109.3 (2)
N3—Cu1—N1	90.32 (7)	N3—C15—C28	128.2 (2)
N3—Cu1—Cl2	110.56 (6)	N4—C15—C28	122.5 (2)
C1—N1—Cu1	125.46 (15)	C17—C16—C1	120.7 (2)
C1—N1—C7	108.16 (18)	C21—C16—C1	119.2 (2)
C7—N1—Cu1	125.71 (14)	C21—C16—C17	120.0 (2)
N1—C1—N2	109.51 (19)	C16—C17—H17	121.2
N1—C1—C16	125.6 (2)	C18—C17—C16	117.6 (2)
N2—C1—C16	124.88 (19)	C18—C17—H17	121.2
C1—N2—C2	130.42 (19)	F1—C18—C17	118.3 (2)
C1—N2—C6	107.59 (17)	F1—C18—C19	118.2 (2)
C2—N2—C6	121.66 (19)	C19—C18—C17	123.6 (2)
N2—C2—H2	120.4	C18—C19—H19	120.9
C3—C2—N2	119.2 (2)	C18—C19—C20	118.2 (2)
C3—C2—H2	120.4	C20—C19—H19	120.9
C9—N3—Cu1	126.13 (15)	C19—C20—H20	119.7
C15—N3—Cu1	125.70 (16)	C19—C20—C21	120.5 (2)
C15—N3—C9	108.14 (19)	C21—C20—H20	119.7
C2—C3—H3	119.5	C16—C21—H21	120.0
C2—C3—C4	121.0 (2)	C20—C21—C16	120.0 (2)
C4—C3—H3	119.5	C20—C21—H21	120.0
C14—N4—C10	122.1 (2)	C23—C22—C8	120.7 (2)
C15—N4—C10	108.36 (18)	C23—C22—C27	119.4 (2)
C15—N4—C14	129.5 (2)	C27—C22—C8	119.9 (2)
C3—C4—H4	119.8	C22—C23—H23	120.8
C5—C4—C3	120.5 (2)	C24—C23—C22	118.5 (2)
C5—C4—H4	119.8	C24—C23—H23	120.8
C4—C5—H5	120.2	F2—C24—C23	118.5 (3)
C4—C5—C6	119.5 (2)	F2—C24—C25	117.9 (3)
C6—C5—H5	120.2	C25—C24—C23	123.6 (3)
N2—C6—C5	118.08 (19)	C24—C25—H25	121.1
C7—C6—N2	106.10 (18)	C24—C25—C26	117.7 (3)
C7—C6—C5	135.8 (2)	C26—C25—H25	121.1
N1—C7—C6	108.62 (18)	C25—C26—H26	119.6
N1—C7—C8	125.69 (19)	C25—C26—C27	120.7 (3)
C6—C7—C8	125.6 (2)	C27—C26—H26	119.6
C7—C8—H8	105.6	C22—C27—H27	119.9
C7—C8—C9	115.73 (18)	C26—C27—C22	120.1 (3)
C7—C8—C22	112.84 (18)	C26—C27—H27	119.9
C9—C8—H8	105.6	C29—C28—C15	119.1 (2)
C9—C8—C22	110.64 (18)	C33—C28—C15	121.2 (2)
C22—C8—H8	105.6	C33—C28—C29	119.5 (3)
N3—C9—C8	125.95 (19)	C28—C29—H29	120.8
C10—C9—N3	108.66 (19)	C30—C29—C28	118.4 (3)
C10—C9—C8	125.3 (2)	C30—C29—H29	120.8
N4—C10—C11	118.2 (2)	F3—C30—C29	117.6 (3)
C9—C10—N4	105.58 (19)	C31—C30—F3	118.8 (3)
C9—C10—C11	136.2 (2)	C31—C30—C29	123.5 (3)
C10—C11—H11	120.5	C30—C31—H31	120.9
C12—C11—C10	119.0 (3)	C30—C31—C32	118.1 (3)
C12—C11—H11	120.5	C32—C31—H31	120.9
C11—C12—H12	119.4	C31—C32—H32	119.9
C11—C12—C13	121.2 (3)	C31—C32—C33	120.2 (3)
C13—C12—H12	119.4	C33—C32—H32	119.9
C12—C13—H13	119.6	C28—C33—C32	120.3 (3)
C14—C13—C12	120.8 (3)	C28—C33—H33	119.9
C14—C13—H13	119.6	C32—C33—H33	119.9
			
Cu1—N1—C1—N2	−171.40 (14)	C7—C8—C22—C27	−113.8 (2)
Cu1—N1—C1—C16	9.1 (3)	C8—C9—C10—N4	178.2 (2)
Cu1—N1—C7—C6	171.14 (14)	C8—C9—C10—C11	−1.9 (4)
Cu1—N1—C7—C8	−10.9 (3)	C8—C22—C23—C24	175.8 (2)
Cu1—N3—C9—C8	−0.3 (3)	C8—C22—C27—C26	−175.7 (2)
Cu1—N3—C9—C10	177.62 (15)	C9—N3—C15—N4	0.6 (3)
Cu1—N3—C15—N4	−177.56 (14)	C9—N3—C15—C28	−177.5 (2)
Cu1—N3—C15—C28	4.4 (4)	C9—C8—C22—C23	−62.1 (3)
F1—C18—C19—C20	178.3 (3)	C9—C8—C22—C27	114.8 (2)
N1—C1—N2—C2	−172.9 (2)	C9—C10—C11—C12	−179.4 (3)
N1—C1—N2—C6	0.4 (2)	C10—N4—C14—C13	0.2 (4)
N1—C1—C16—C17	−129.4 (3)	C10—N4—C15—N3	−0.4 (3)
N1—C1—C16—C21	48.9 (3)	C10—N4—C15—C28	177.7 (2)
N1—C7—C8—C9	35.2 (3)	C10—C11—C12—C13	−0.8 (4)
N1—C7—C8—C22	−93.7 (3)	C11—C12—C13—C14	0.9 (5)
C1—N1—C7—C6	0.1 (2)	C12—C13—C14—N4	−0.5 (4)
C1—N1—C7—C8	178.1 (2)	C14—N4—C10—C9	179.8 (2)
C1—N2—C2—C3	174.9 (2)	C14—N4—C10—C11	−0.1 (3)
C1—N2—C6—C5	−177.90 (19)	C14—N4—C15—N3	179.9 (2)
C1—N2—C6—C7	−0.4 (2)	C14—N4—C15—C28	−1.9 (4)
C1—C16—C17—C18	178.3 (2)	C15—N3—C9—C8	−178.4 (2)
C1—C16—C21—C20	−178.4 (2)	C15—N3—C9—C10	−0.5 (3)
F2—C24—C25—C26	179.6 (3)	C15—N4—C10—C9	0.1 (2)
N2—C1—C16—C17	51.1 (3)	C15—N4—C10—C11	−179.8 (2)
N2—C1—C16—C21	−130.6 (2)	C15—N4—C14—C13	179.8 (3)
N2—C2—C3—C4	0.9 (4)	C15—C28—C29—C30	175.7 (2)
N2—C6—C7—N1	0.1 (2)	C15—C28—C33—C32	−175.7 (3)
N2—C6—C7—C8	−177.80 (19)	C16—C1—N2—C2	6.7 (4)
C2—N2—C6—C5	−3.9 (3)	C16—C1—N2—C6	180.0 (2)
C2—N2—C6—C7	173.66 (19)	C16—C17—C18—F1	−178.8 (2)
C2—C3—C4—C5	−2.6 (4)	C16—C17—C18—C19	0.6 (4)
F3—C30—C31—C32	−178.9 (3)	C17—C16—C21—C20	−0.1 (4)
N3—C9—C10—N4	0.3 (2)	C17—C18—C19—C20	−1.1 (4)
N3—C9—C10—C11	−179.9 (3)	C18—C19—C20—C21	1.0 (4)
N3—C15—C28—C29	85.9 (3)	C19—C20—C21—C16	−0.4 (4)
N3—C15—C28—C33	−98.4 (3)	C21—C16—C17—C18	0.0 (4)
C3—C4—C5—C6	1.0 (3)	C22—C8—C9—N3	100.8 (2)
N4—C10—C11—C12	0.4 (3)	C22—C8—C9—C10	−76.8 (3)
N4—C15—C28—C29	−91.9 (3)	C22—C23—C24—F2	−179.0 (2)
N4—C15—C28—C33	83.8 (3)	C22—C23—C24—C25	0.3 (4)
C4—C5—C6—N2	2.1 (3)	C23—C22—C27—C26	1.2 (4)
C4—C5—C6—C7	−174.5 (2)	C23—C24—C25—C26	0.3 (4)
C5—C6—C7—N1	177.0 (2)	C24—C25—C26—C27	−0.2 (4)
C5—C6—C7—C8	−0.9 (4)	C25—C26—C27—C22	−0.6 (4)
C6—N2—C2—C3	2.4 (3)	C27—C22—C23—C24	−1.0 (4)
C6—C7—C8—C9	−147.2 (2)	C28—C29—C30—F3	179.2 (3)
C6—C7—C8—C22	83.9 (3)	C28—C29—C30—C31	−0.1 (5)
C7—N1—C1—N2	−0.3 (2)	C29—C28—C33—C32	0.1 (5)
C7—N1—C1—C16	−179.9 (2)	C29—C30—C31—C32	0.4 (5)
C7—C8—C9—N3	−29.1 (3)	C30—C31—C32—C33	−0.4 (5)
C7—C8—C9—C10	153.3 (2)	C31—C32—C33—C28	0.2 (5)
C7—C8—C22—C23	69.4 (3)	C33—C28—C29—C30	−0.1 (4)

### Dichlorido{1,1'-[(3-fluorophenyl)methylene]bis[3-(3-fluorophenyl)imidazo[1,5-a]pyridine]-κ2N,N'}copper(II) Hydrogen-bond geometry (Å, º)

*Cg*1, *Cg*2 and *Cg*3 are the centroids of rings N2/C2–C6, N3/N4/C9–C10/C15 and N4/C10–C14, respectively.

**Table:**

*D*—H···*A*	*D*—H	H···*A*	*D*···*A*	*D*—H···*A*
C12—H12···F3^i^	0.93	2.47	3.384 (4)	166
C23—H23···Cl2	0.93	2.81	3.736 (3)	174
C20—H20···*Cg*1^ii^	0.93	2.77	3.477 (3)	133
C18—F1···*Cg*2^iii^	1.35 (1)	3.23 (1)	4.484 (3)	154 (1)
C24—F2···*Cg*3^iv^	1.36 (1)	3.57 (1)	4.284 (3)	113 (1)

Symmetry codes: (i) *x*, *y*+1, *z*; (ii) −*x*, −*y*+1, −*z*+1; (iii) −*x*+1, −*y*+1, −*z*+1; (iv) *x*−1, *y*, *z*.
