# Supplementary material for: Syntheses, structures and anti­cancer activities of CuII and ZnII complexes containing 1,1′-[(3-fluoro­phen­yl)methyl­ene]bis­[3-(3-fluoro­phen­yl)imidazo[1,5-a]pyridine]
Source: Acta Crystallogr E Crystallogr Commun. 2025 Jan 1;81(Pt 1):47–52. doi: 10.1107/S2056989024011617 (PMC11701767; doi:10.1107/S2056989024011617)
Supplement: Supplementary file 3 [file e-81-00047-sup3.docx]

**Synthesis, structure and anticancer activities of Cu(II) and Zn(II) complexes containing 1,1'-((3-fluorophenyl)methylene)bis(3-(3-fluorophenyl)imidazo[1,5-*a*]pyridine**

Le Thi Hong Hai^a,b^, Nguyen Van Hau^a^, Hoang Tuan Duong^a^, Tran Quang Hung^c^, Dang Thanh Tuan^c^,
Luc Van Meervelt^d^*

^a^Department of Chemistry, Hanoi National University of Education, 136 Xuan Thuy, Cau Giay, Hanoi, Vietnam.

^b^Institute of Natural Sciences, Hanoi National University of Education, 136 Xuan Thuy, Cau Giay, Hanoi, Vietnam

^c^Institute of Chemistry, Vietnamese Academy of Science and Technology (VAST),18 Hoang Quoc Viet, Hanoi, Vietnam

^d^Department of Chemistry, KU Leuven, Biomolecular Architecture, Celestijnenlaan 200F, Leuven (Heverlee), B-3001, Belgium

Correspondence e-mail: [hailth@hnue.edu.vn](mailto:hailth@hnue.edu.vn), [luc.vanmeervelt@kuleuven.be](mailto:luc.vanmeervelt@kuleuven.be)

**Supporting information**

Figure S1. IR spectra of **T4**

Figure S2. ^1^H NMR spectra of **T4**

Figure S3. Positive mass spectrum of **CuT4** complex

Figure S4. Negative mass spectrum of **CuT4** complex

Figure S5. IR spectra of **CuT4** complex

Figure S6. Picture of the **CuT4** crystals

Figure S7. Positive mass spectra of **ZnT4** complex

Figure S8. IR spectra of **ZnT4** complex

Figure S9. ^1^H NMR spectra of **ZnT4** complex


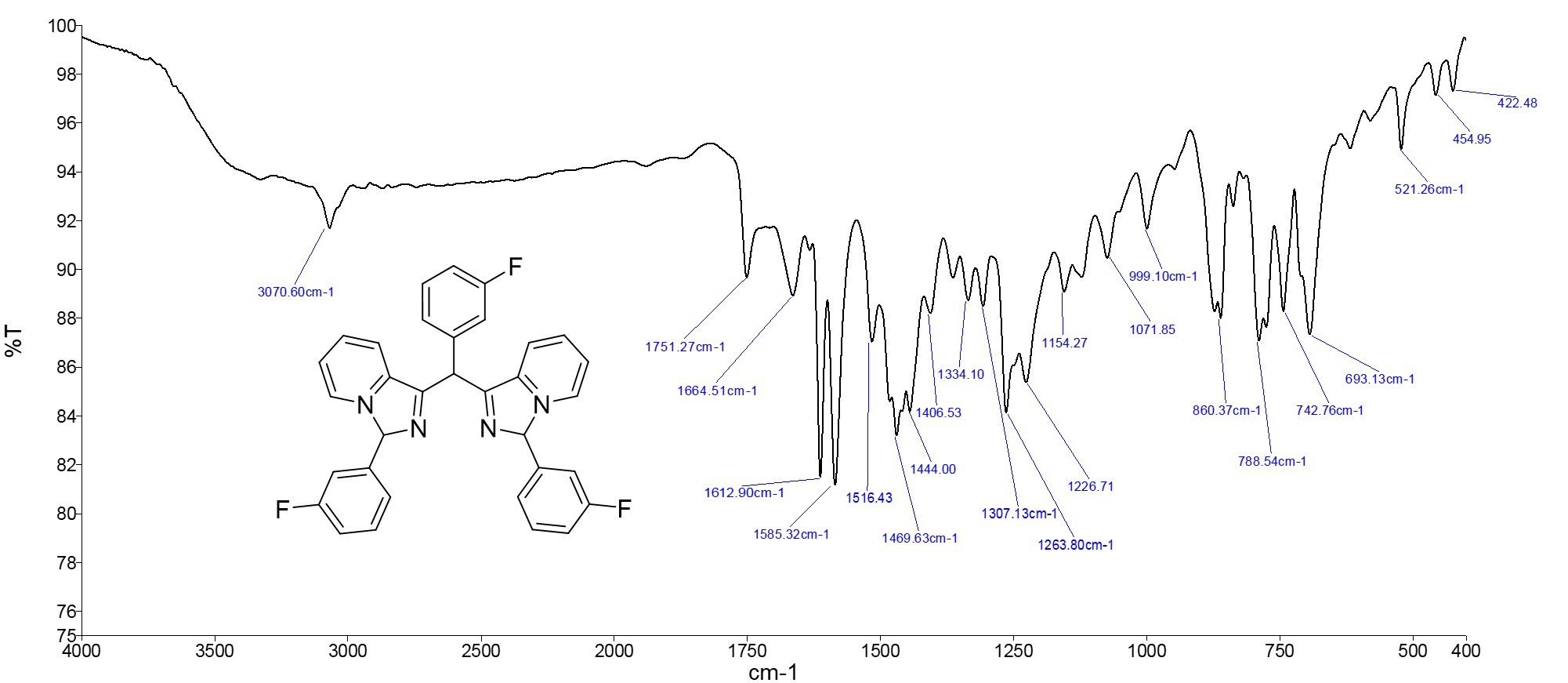


Figure S1. IR spectra of **T4**


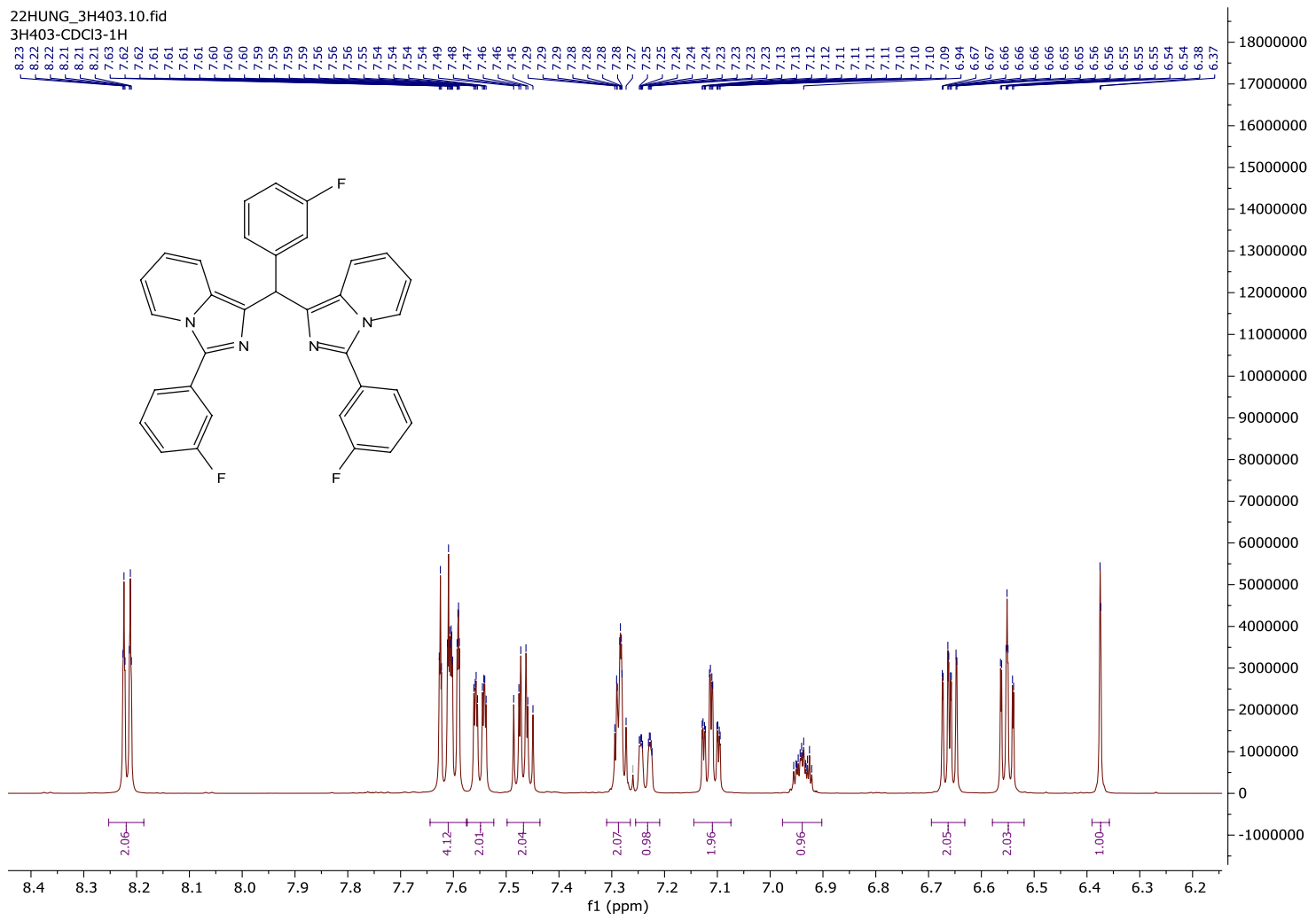


Figure S2. ^1^H NMR spectra of **T4**


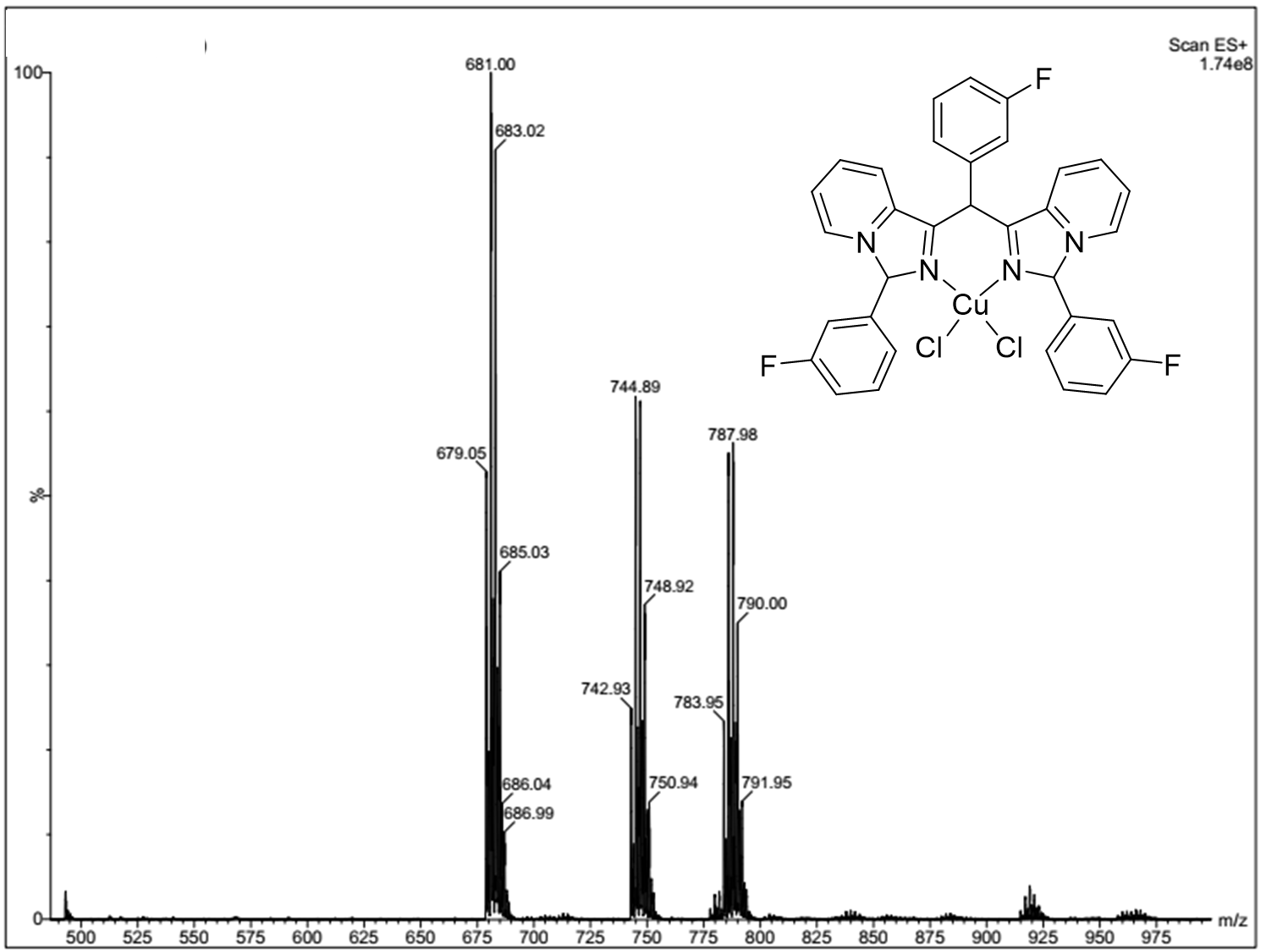


Figure S3. Positive mass spectrum of **CuT4** complex


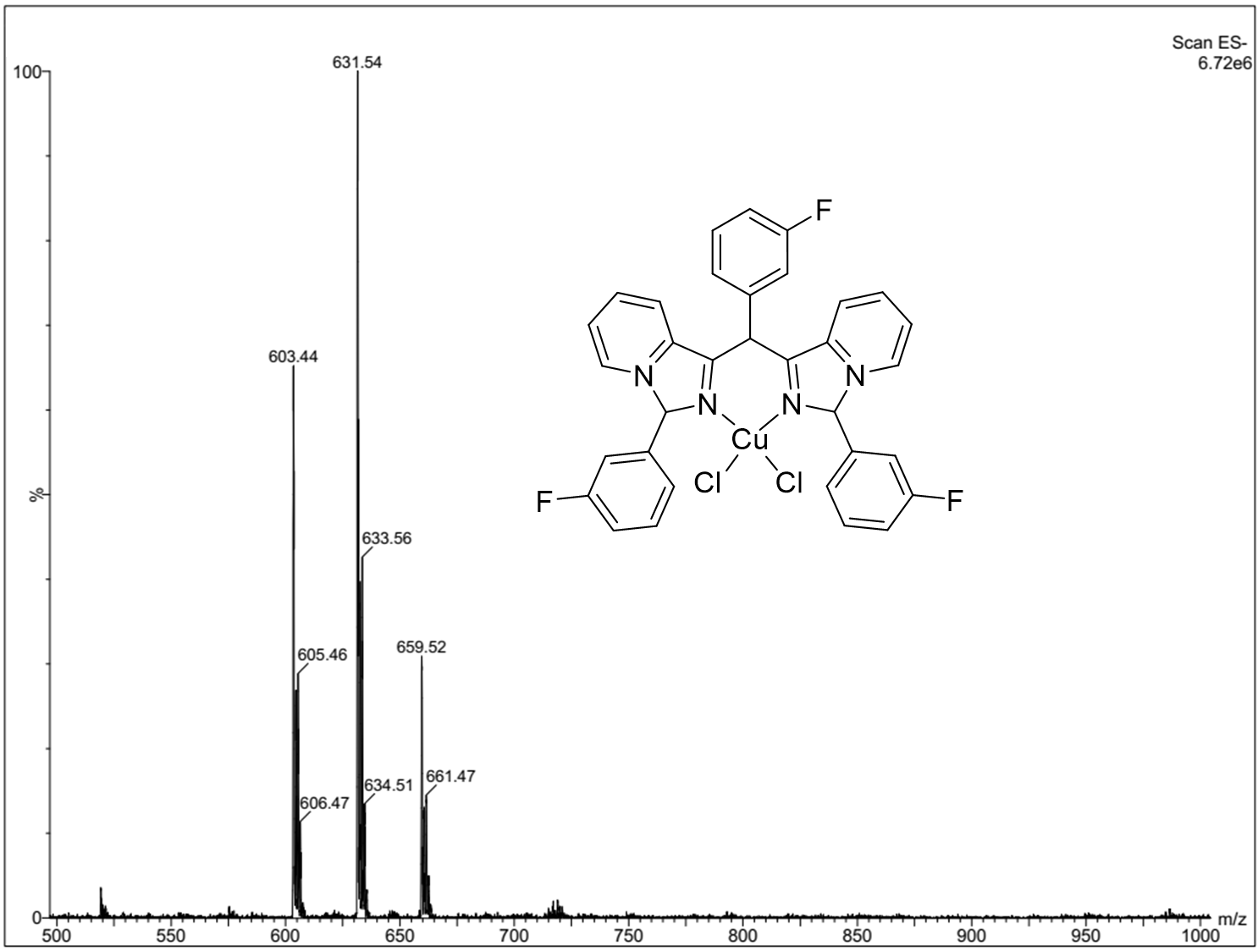


Figure S4. Negative mass spectrum of **CuT4** complex


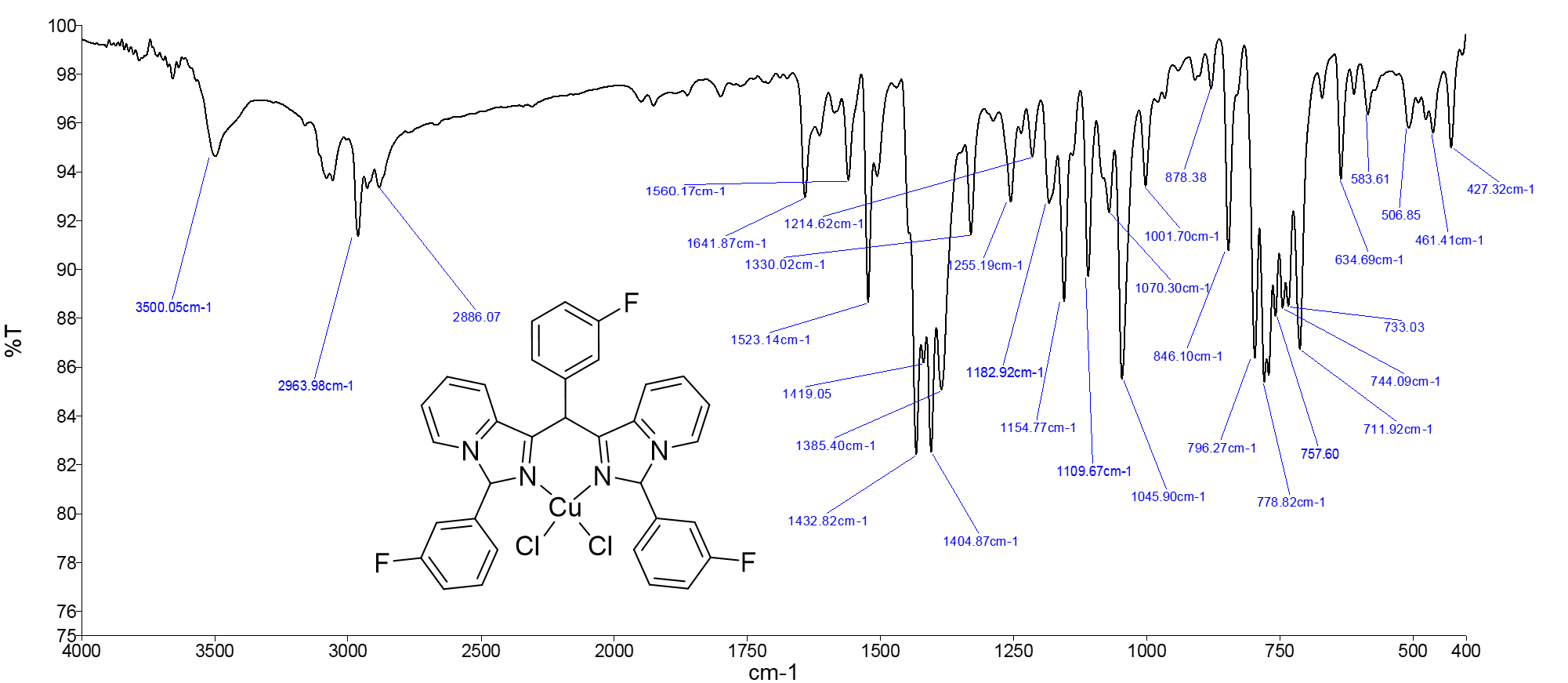


Figure S5. IR spectra of **CuT4** complex


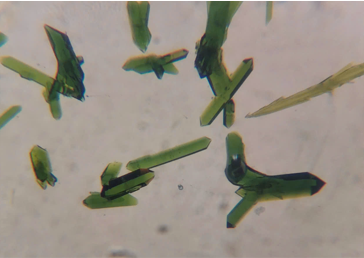


Figure S6. Picture of the **CuT4** crystals


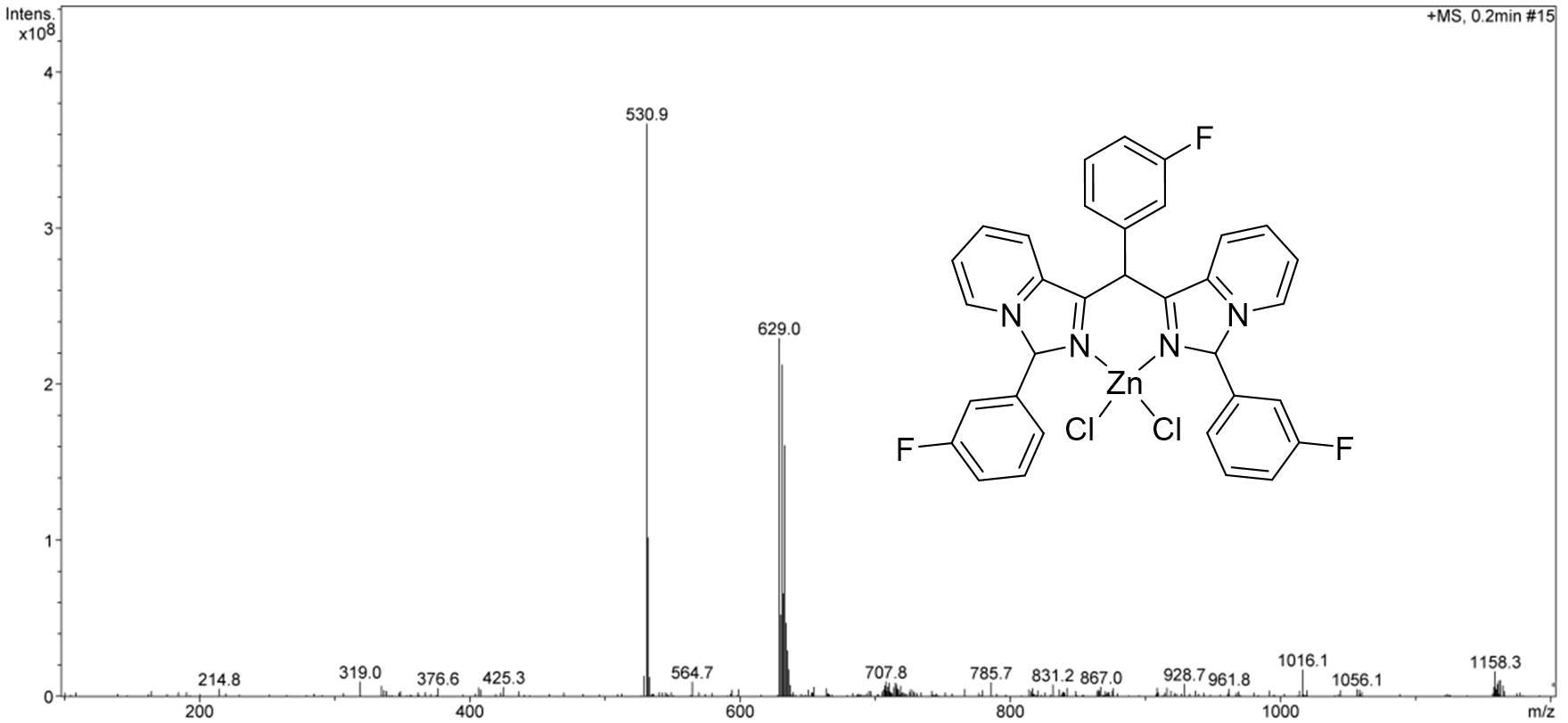


Figure S7. Positive mass spectra of **ZnT4** complex


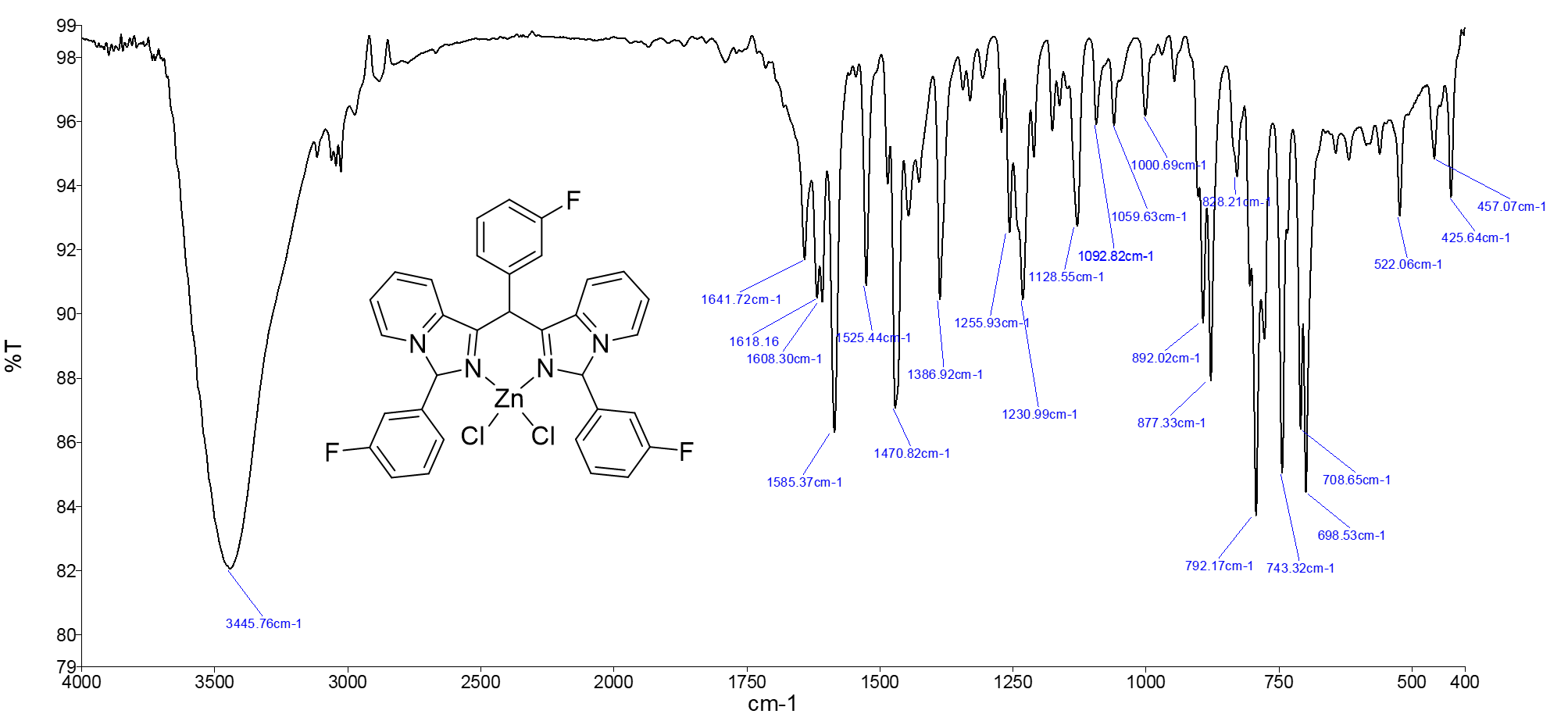


Figure S8. IR spectra of **ZnT4** complex


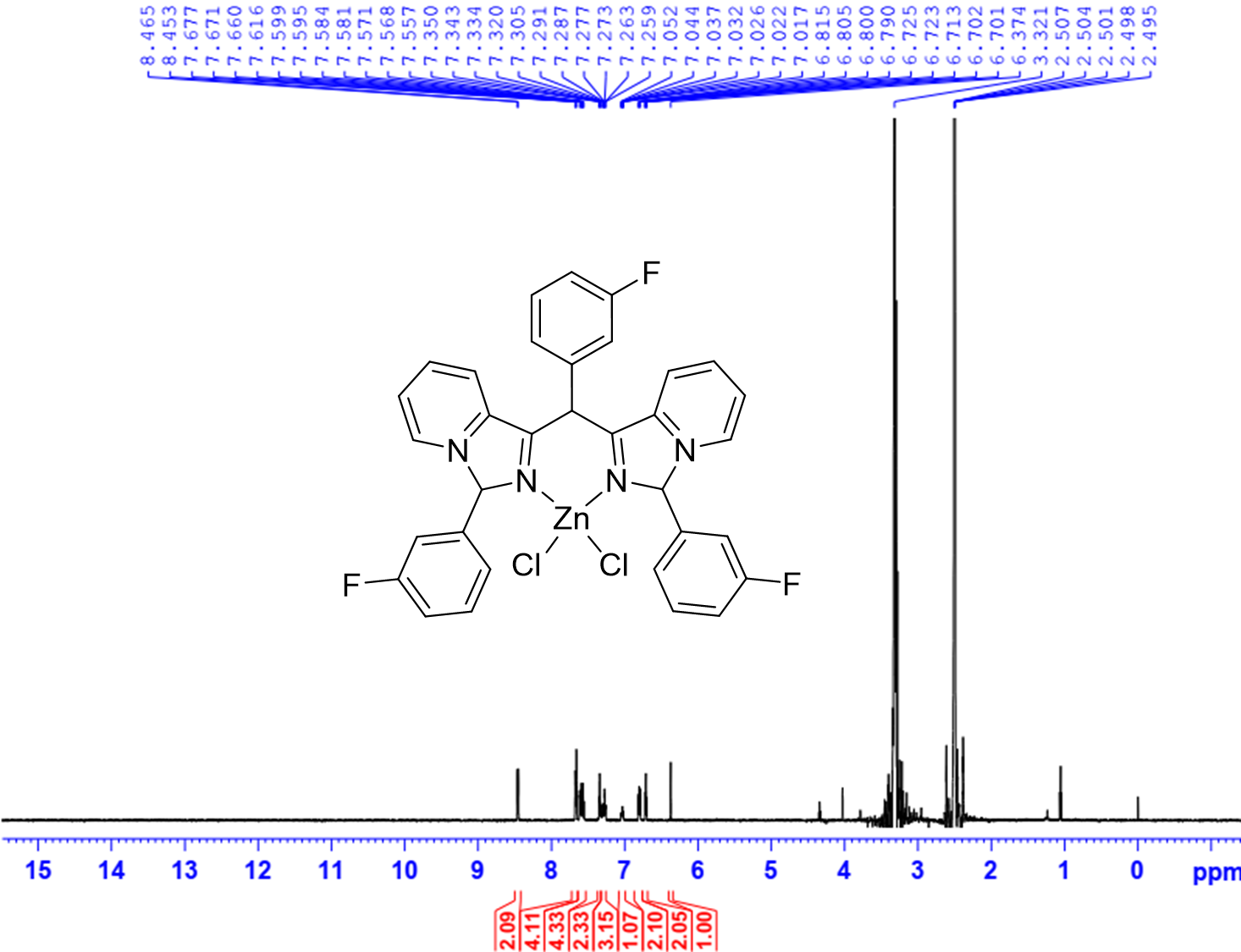


Figure S9. ^1^H NMR spectra of **ZnT4** complex
